# A ventral pallidal glutamatergic aversive network encodes abstinence from and reexposure to cocaine

**DOI:** 10.1126/sciadv.adu6074

**Published:** 2025-07-23

**Authors:** Liran A. Levi, Kineret Inbar, Esti Tseiger, Yonatan M. Kupchik

**Affiliations:** ^1^ Department of Medical Neurobiology, Institute for Medical Research Israel-Canada (IMRIC), Faculty of Medicine, The Hebrew University of Jerusalem, Jerusalem, Israel.; ^2^ IMRIC Center for Addiction Research (ICARe), The Hebrew University of Jerusalem, Jerusalem, Israel.

## Abstract

Relapse to drugs of abuse can occur after long periods of abstinence. The ventral pallidum (VP) is central to drug addiction, and its glutamatergic neurons (VP_Glu_), whose activation drives aversion, inhibit drug seeking. However, it remains unknown whether these neurons encode the abstinence from and relapse to drugs. We show here that VP_Glu_ projections specifically to the aversion-related lateral habenula (LHb) and ventral tegmental area gabaergic (VTA_GABA_) neurons show plasticity induced by abstinence from and reexposure to cocaine or cocaine cues. Both these pathways potentiate during abstinence and restore baseline values upon drug reexposure but with different plasticity mechanisms. Last, inhibiting the VP_Glu_ → LHb pathway enhances cocaine preference after abstinence, while inhibiting the VP_Glu_ → VTA pathway shows variable effects. These findings establish an aversive circuit orchestrated by VP_Glu_ neurons encoding long-term abstinence-driven changes that may contribute to drug relapse.

## Teaser

Cocaine abstinence and reexposure generate permanent changes in specific ventral pallidal glutamatergic outputs.

## INTRODUCTION

A central issue in drug addiction is the high rates of relapse, driven by drug craving (*1*) and the negative emotions induced by withdrawal (*2*). Neural adaptations during withdrawal were primarily shown in the nucleus accumbens and the ventral tegmental area (VTA) (*3*–*7*) and linked mainly to the growing craving for the drug. Adaptations underlying the contribution of aversive symptoms to relapse, however, are less well understood.

The ventral pallidum (VP) is a central structure within the basal ganglia (*8*, *9*) composed primarily of GABAergic neurons whose activation generates conditioned place preference (CPP) and reward seeking (*10*, *11*) and is linked to drug seeking (*12*, *13*). In addition to the GABAergic neurons, 10 to 15% of VP neurons (*8*, *14*, *15*) express the vesicular glutamatergic transporter 2 (vGluT2) and are considered glutamatergic neurons. The glutamatergic neurons of the ventral pallidum (VP_Glu_) are more active during aversive experiences (*10*) and induce conditioned place aversion, presumably by activating the lateral habenula (LHb), a major center of aversion (*11*, *14*). VP_Glu_ neurons project to many downstream targets of the VP, and we have shown that they are strategically connected more strongly with neurons that induce aversion, such as the LHb and VTA GABAergic (VTA_GABA_) neurons, while showing weaker synaptic connections with reward-inducing targets, such as VTA dopamine (VTA_DA_) and VP GABAergic (VP_GABA_) neurons (*15*). Thus, these neurons may be involved in withdrawal or abstinence from drugs. Recent studies show that the activity of VP_Glu_ neurons, particularly that of VP_Glu_ neurons projecting to the LHb (VP_Glu_ → LHb), correlates with withdrawal from drugs and their activation inhibits drug seeking (*13*, *16*). Nevertheless, it remains unknown whether abstinence from drugs generates long-term adaptations in VP_Glu_ neurons, particularly at the synaptic level. More specifically, it is not known whether prolonged abstinence induces long-term synaptic plasticity in VP_Glu_ outputs, whether such plasticity is general or restricted to specific outputs, and whether terminating abstinence by reexposure to the drug, an event that presumably diminishes withdrawal symptoms, affects synaptic outputs of VP_Glu_ neurons.

In this work, we provide a comprehensive dissection of five VP_Glu_ synapses—on LHb, VTA_GABA_, VTA_DA_, VP_GABA_, or other VP_Glu_ neurons—throughout the process of abstinence and reexposure to cocaine. We show that abstinence drives plasticity in VP_Glu_ synapses selectively onto the aversion-inducing LHb and VTA_GABA_ neurons but with different mechanisms in each pathway.

## RESULTS

### VP_Glu_ neurons are central in cocaine CPP after prolonged abstinence

**Fig. 1.**
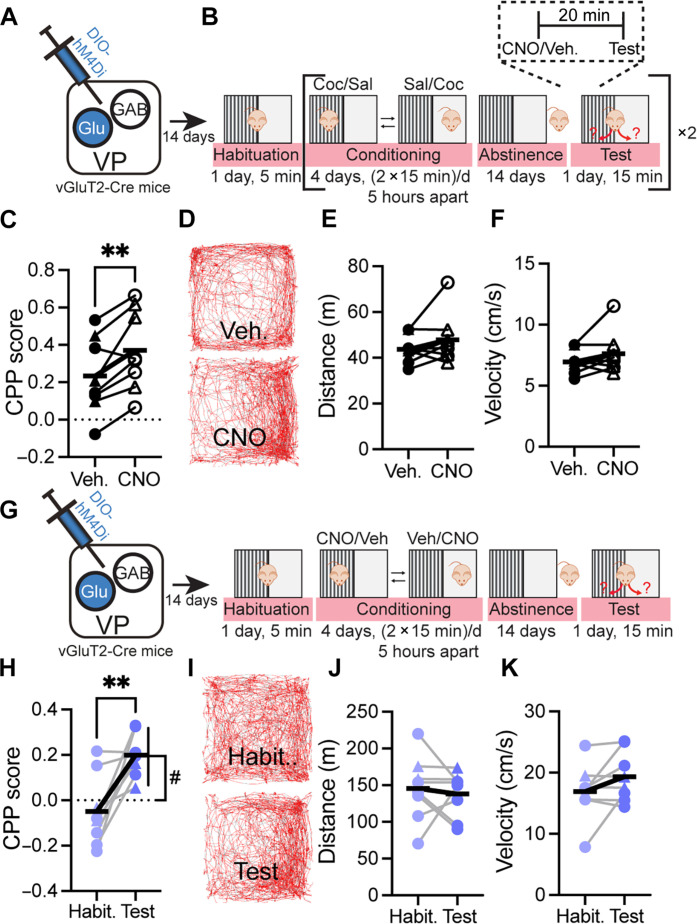
Inhibition of VP_Glu_ neurons is rewarding and enhances cocaine CPP after abstinence. Circles, females; triangles, males. Horizontal bold lines represent group averages. (**A** to **F**) Inhibition of VP_Glu_ neurons during the cocaine CPP test. (A) Microinjection strategy. (B) Experimental protocol. Mice underwent the CPP test 14 days after cocaine conditioning. We injected mice with CNO (3 mg/kg, ip) or vehicle (Veh.) 20 min before test. Mice were then reconditioned, went through abstinence, and tested again but injected with vehicle/CNO, respectively. Order of CNO/vehicle injections was counterbalanced to avoid order effects. (C) Inhibiting VP_Glu_ neurons enhanced the CPP score from 0.23 ± 0.2 to 0.37 ± 0.2 (**, paired *t* test; *t*_7_ = 3.53, *P* = 0.0097). (CPP = 0 represents indifference). (D) Trajectory movement maps of a mouse injected with vehicle (top) or CNO (bottom). (E and F) VP_Glu_ inhibition did not affect the distance covered (E) (43.7 ± 6.2 m and 47.9 ± 11.1 m with saline/CNO injections, respectively) or the velocity of mice (F) (6.96 ± 1.00 cm/s and 7.62 ± 1.74 cm/s with saline/CNO injections, respectively) (paired *t* tests). (**G** to **K**) Conditioning to the inhibition of VP_Glu_ neurons. (G) Microinjection strategy, same as in (A), and experimental protocol—mice expressing hM4Di in VP_Glu_ neurons were injected with CNO (3 mg/kg, ip) in one side of the CPP box and vehicle in the other side of the box. (H) Mice showed preference for the side paired with VP_Glu_ inhibition [CPP_Habituation_ = −0.05 ± 0.2, CPP_Test_ = 0.20 ± 0.1; **, paired *t* test; *t*_7_ = 3.65, *P* = 0.0082; #, CPP_Test_ compared to zero (indifference), one-sample *t* test; *t*_7_ = 5.93, *P* = 0.0006]. (I) Trajectory movement maps of a mouse during habituation (top) and CPP test (bottom). (J and K) VP_Glu_ inhibition during conditioning did not affect the distance covered (145.3 ± 44.5 m and 138.1 ± 30.7 m at habituation and CPP test, respectively) (J) or the velocity of movement (16.90 ± 4.63 cm/s and 19.33 ± 4.12 cm/s at habituation and CPP test, respectively) (K) (paired *t* tests). All groups consist of eight mice.

Activation of VP_Glu_ neurons generates aversion (*11*, *14*, *17*, *18*) and inhibits heroin taking (*16*), and the synapses these neurons make on classical aversive targets, such as the LHb or VTA_GABA_ neurons, are potentiated after abstinence from cocaine (*15*). We therefore wanted to examine whether inhibition of VP_Glu_ neurons affected cocaine CPP after abstinence. We injected vGluT2-Cre mice with an adeno-associated virus (AAV) harboring a DNA sequence encoding for the inhibitory Gi-coupled designer receptors exclusively activated by designer drugs (DREADDs) in a double-inverted orientation (DIO) (AAV-DIO-hM4Di-mCherry) in the VP to infect VP_Glu_ neurons (Fig. 1A and fig. S1). After recovery, mice were first conditioned to receive cocaine in a specific side of a two-chamber box and then underwent 14 days of abstinence. After abstinence, each mouse went through two CPP tests—with intraperitoneal injection of clozapine-N-oxide (CNO; 3 mg/kg) or vehicle 20 min before the test (injection order counterbalanced). The tests were separated by 4 days of reconditioning (two sessions/day) followed by 14 additional days of abstinence (Fig. 1B). Inhibiting the VP_Glu_ neurons generated an increase in cocaine CPP (Fig. 1, C and D)—it elevated the average CPP score from 0.23 ± 0.2 to 0.37 ± 0.08 (*P* < 0.01), an average increase of 0.14 ± 0.11 in the CPP score (fig. S2)—without affecting locomotion during the CPP test (Fig. 1, E and F; see full statistical information in table S1). Expectedly, injection of cocaine during the conditioning sessions did increase locomotion while cocaine was on board (fig. S3).

Considering the known role of VP_Glu_ neurons in generating aversion (*11*, *14*, *17*, *18*), the data raise the possibility that inhibition of VP_Glu_ neurons may induce reward, or rather inhibit aversion, that is capable of priming and enhancing cocaine CPP. To further examine this, we expressed AAV-DIO-hM4Di-mCherry in VP_Glu_ neurons as described above and used the CPP protocol to condition the inhibition of VP_Glu_ neurons to a specific side of the box (Fig. 1G). In CPP tests performed 14 days after the last conditioning session, mice showed clear preference for the side paired with VP_Glu_ inhibition—the CPP score was 0.2 ± 0.09 compared to −0.05 ± 0.15 before conditioning (Fig. 1, H and I), reflecting an average increase of 0.25 ± 0.19 in the CPP score (fig. S2). Thus, inhibition of VP_Glu_ neurons does not only enhance cocaine CPP but is rewarding in itself. As in the cocaine CPP experiments, inhibition of VP_Glu_ neurons during conditioning did not affect locomotion during the CPP test (Fig. 1, J and K). However, inhibition of the VP_Glu_ neurons did enhance locomotion while CNO was on board in the conditioning session (fig. S3), although not as strongly as cocaine did. Thus, inhibition of VP_Glu_ neurons and cocaine injections both generate preference and enhance locomotion while on board.

### Postsynaptic plasticity in VP_Glu_ → LHb synapses encodes cocaine abstinence and reexposure

Our behavioral data show a clear involvement of VP_Glu_ neurons in the expression of cocaine CPP after abstinence. However, it does not imply the involvement of specific VP_Glu_ projections. Our previous work showed that VP_Glu_ neurons synapse more strongly on aversion-related targets like LHb and VTA_GABA_ neurons (*15*). Therefore, we next examined whether these “aversive” outputs of VP_Glu_ neurons, in comparison to other VP_Glu_ projections, encode the condition of the mouse throughout the CPP process more reliably than other VP_Glu_ outputs. We hypothesized that VP_Glu_ synapses that are relevant for the encoding of prolonged abstinence from and reexposure to cocaine would change dynamically during these phases to reflect the condition of the mouse.

**Fig. 2.**
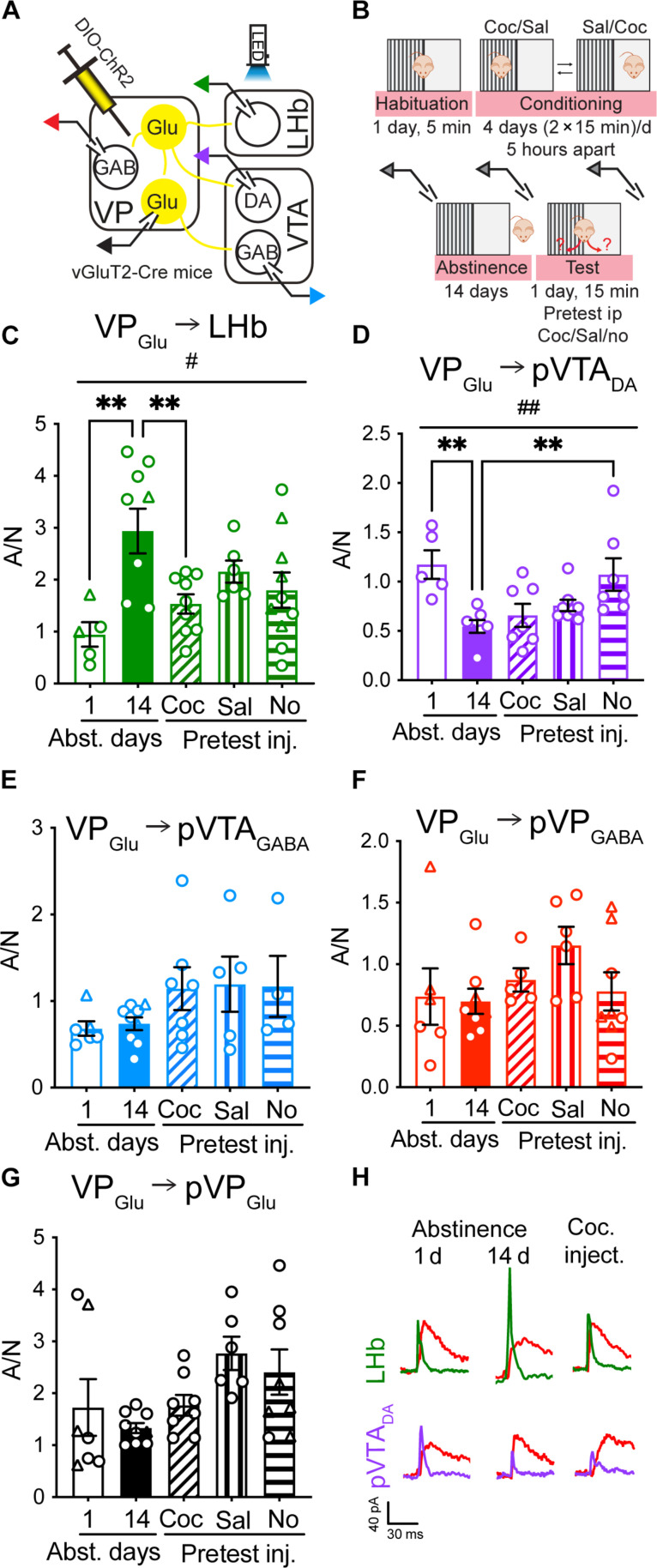
VP_Glu_ → LHb neurons show postsynaptic plasticity that tracks abstinence from and reexposure to cocaine. (**A**) Viral injection and recording strategy. We injected AAV-DIO-ChR2-eYFP into the VP of vGluT2-Cre mice and activated VP_Glu_ terminals optogenetically while recording from pVP_Glu_, pVP_GABA_, pVTA_GABA_, pVTA_DA_, or LHb neurons. (**B**) Experimental protocol. Mice were euthanized for recording at three time points—after acute (1 day) abstinence, after prolonged (14 days) abstinence, or after a 15-min CPP test preceded by reexposure to either cocaine (15 mg/kg, ip), vehicle, or no injection. (C to G) Circles/triangles, cells taken from females/males, respectively. (**C**) A/N ratios in VP_Glu_ → LHb synapses at all five conditions. Prolonged abstinence increased the A/N from 0.94 ± 0.5 in acute abstinence to 2.94 ± 1.3 [one-way ANOVA, *F*_(4,34)_ = 4.70, *P* = 0.004, Dunnett’s multiple comparisons test, *P* = 0.002] and reexposure to cocaine after prolonged abstinence reduced the A/N (*P* = 0.009 comparing to 14 days of abstinence) back to baseline levels (1.53 ± 0.6, *P* = 0.76 comparing to the first day of abstinence). (**D**) Prolonged abstinence from cocaine decreased the A/N ratio in the VP_Glu_ → pVTA_DA_ synapse from 1.17 ± 0.3 to 0.55 ± 0.2 [one-way ANOVA, *F*_(4,29)_ = 5.24, *P* = 0.003, Dunnett’s multiple comparisons test, *P* = 0.02]. The low A/N was not changed by reexposure to cocaine or a vehicle injection but was increased back to the baseline level after a CPP test with no pretest injection (*P* = 0.008). (**E** to **G**) Abstinence or reexposure to cocaine did not change the A/N in the VP_Glu_ → pVTA_GABA_ (E), VP_Glu_ → pVP_GABA_ (F), and VP_Glu_ → pVP_Glu_ (G) synapses. A pretest saline injection increased the A/N in the VP_Glu_ → pVP_Glu_ synapse from 1.33 ± 0.29 after prolonged abstinence to 2.77 ± 0.79, but this did not reach significance (*P* = 0.024, significance threshold is *P* = 0.0167 after Bonferroni correction for multiple comparisons; see Methods). (**H**) Representative traces of A/N recordings from the VP_Glu_ → LHb and VP_Glu_ → pVTA_DA_ synapses after acute and prolonged abstinence and after reexposure to cocaine. Number of cells ranged between 4 and 10 in all experiments.

To evaluate the synaptic properties of five VP_Glu_ outputs—to LHb, VTA_DA_, VTA_GABA_, VP_GABA_, or VP_Glu_ neurons—throughout the entire CPP process, we first expressed channelrhodopsin 2 (ChR2) in VP_Glu_ neurons (Fig. 2A and fig. S1) and then trained mice on the cocaine CPP task, followed by 14 days of abstinence (Fig. 2B). After abstinence we reintroduced mice to the CPP box to examine their preference for the cocaine-paired side. Before the CPP test, mice received a cocaine challenge to terminate the abstinence period. Control mice received a saline challenge or no challenge. All three conditions generated a clear and similar preference for the cocaine-paired side (fig. S4) Whole-cell patch-clamp recordings from acute brain slices were performed at three time points—in acute abstinence (24 hours after the last cocaine injection), after prolonged abstinence (14 days after the last cocaine injection), or 15 min after reexposure to cocaine and the CPP test. We recorded and compared three parameters from each cell and used the Bonferroni method to correct for multiple comparisons. Thus, in these experiments, the threshold for significance is *P* = 0.0167. Putative cell types in the VP (putative VP_Glu_ and VP_GABA_, pVP_Glu_ and pVP_GABA_, respectively) or VTA (pVTA_DA_ and pVTA_GABA_) were determined as described in Methods and in figs. S5 and S6.

We first examined the postsynaptic changes by comparing the ratio between AMPA and *N*-methyl-d-aspartate (NMDA) currents (A/N) in acute and prolonged abstinence from cocaine and after reexposure to cocaine. A one-way analysis of variance (ANOVA) test on each projection revealed that only the synapses on the LHb and VTA_DA_ showed a significant cocaine condition main effect (Fig. 2; see tables S2 and S3 for all statistics). Looking within each projection, our data reveal that the A/N ratio at the VP_Glu_ → LHb synapse changed dynamically during the CPP process—it increased from 0.94 ± 0.5 in acute abstinence to 2.94 ± 1.3 after prolonged abstinence and restored to baseline levels (1.53 ± 0.6) by reexposure to cocaine before a CPP test (Fig. 2, C and H). Putting the mouse through the CPP test but without priming it with cocaine (i.e., with saline injection or no injection) resulted in A/N ratios (2.16 ± 0.5 and 1.8 ± 1.1, respectively) that were higher than those of the cocaine group but lower than the prolonged abstinence group, not significantly different from either. Thus, only reexposure to cocaine and the CPP box was sufficient to restore the acute abstinence A/N values. In addition, the A/N ratios at the VP_Glu_ → LHb synapse were inversely correlated with the CPP score in the three CPP groups combined (fig. S7), suggesting that increased preference is linked to a weaker VP_Glu_ → LHb synapse.

The VP_Glu_ → pVTA_DA_ synapse responded to prolonged abstinence opposite to the VP_Glu_ → LHb response—the A/N in this synapse decreased from 1.17 ± 0.3 to 0.55 ± 0.2 (Fig. 2, D and H). Unlike the VP_Glu_ → LHb synapse, the A/N in the VP_Glu_ → pVTA_DA_ synapse was not affected by reexposure to cocaine or by a saline injection, but reintroduction to the CPP box without any injection was able to restore preabstinence A/N values. There was no significant difference between the three groups undergoing CPP after abstinence [*F*_(2,19)_ = 3.26, *P* = 0.061], and the A/N in this synapse did not correlate with the CPP score (fig. S7). The A/N in the synapses between VP_Glu_ neurons and pVTA_GABA_ (Fig. 2E), pVP_GABA_ (Fig. 2F), or pVP_Glu_ (Fig. 2G) was not affected by prolonged abstinence or reexposure to cocaine or the CPP box and did not correlate with the CPP score (fig. S7), although a pretest injection of saline seemed to increase the A/N in the synapses on VP_Glu_ neurons (did not reach significance). This may indicate that the VP_Glu_ → pVP_Glu_ synapse encodes the aversive effect of the injection.

### VP_Glu_ → LHb and VP_Glu_ → pVTA_GABA_ presynaptic plasticity encodes cocaine abstinence and reexposure

Cocaine abstinence and reexposure may induce not only postsynaptic changes but also plasticity in the release of glutamate from VP_Glu_ terminals. We therefore next aimed to determine whether prolonged abstinence from or reexposure to cocaine altered the probability of glutamate release at the five VP_Glu_ projections examined here.

**Fig. 3.**
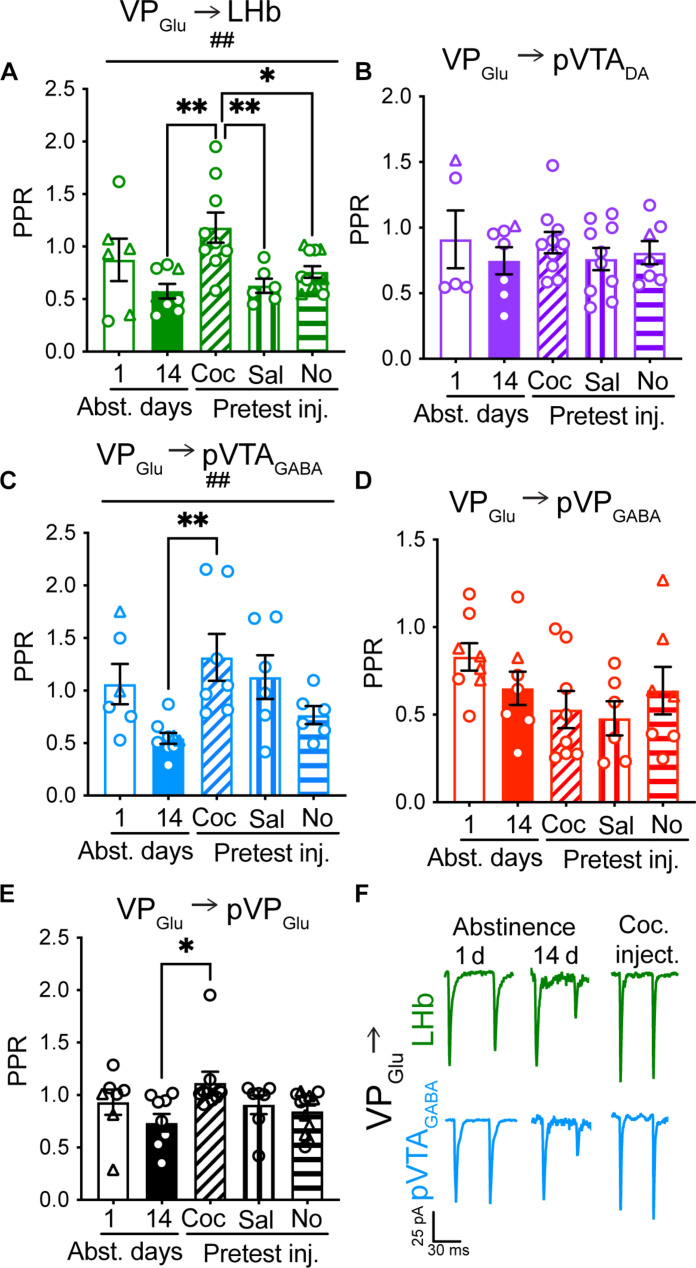
Reexposure to cocaine after prolonged restores baseline PPR in VP_Glu_ synapses on LHb and pVTA_GABA_. (A to E) Circles, cells taken from females; triangles, cells taken from males. (**A** and **C**) The PPR of the VP_Glu_ projections to the LHb (A) and the pVTA_GABA_ (C) changed across the different stages of the CPP protocol [##, one-way ANOVA, VP_Glu_ synapses on LHb: *F*_(4,34)_ = 4.38, *P* = 0.006; pVTA_GABA_: *F*_(4,29)_ = 4.22, *P* = 0.008]. Fourteen days of abstinence after cocaine CPP caused a decrease in the PPR of both VP_Glu_ → LHb (from 0.87 ± 0.49 to 0.61 ± 0.19) and VP_Glu_ → pVTA_GABA_ (from 1.06 ± 0.47 to 0.55 ± 0.16) synapses compared to the first day of abstinence. Reexposure to cocaine after prolonged abstinence significantly increased the PPR in both synapses (**, one-way ANOVA, Dunnett’s multiple comparisons test, *P* = 0.004 for VP_Glu_ → LHb and *P* = 0.003 for VP_Glu_ → pVTA_GABA_). In VP_Glu_ → pVTA_GABA_ synapses a pretest saline injection also increased the PPR compared to after 14 days of abstinence (*P = 0.04). (**B** and **D**) Cocaine abstinence or reexposure did not affect the PPR of the VP_Glu_ synapses on pVTA_DA_ or pVP_GABA_ neurons. (**E**) Reexposure to cocaine after 14 days of abstinence increased the PPR in the VP_Glu_ → pVP_Glu_ synapse (*, one-way ANOVA, Dunnett’s multiple comparisons test, *P* = 0.01). (**F**) Representative traces of consecutive currents recorded in VP_Glu_ → LHB and VP_Glu_ → pVTA_GABA_ synapses after 1 and 14 days of abstinence and after reexposure to cocaine. Number of cells ranged between 5 and 12 in all experiments.

**Fig. 4.**
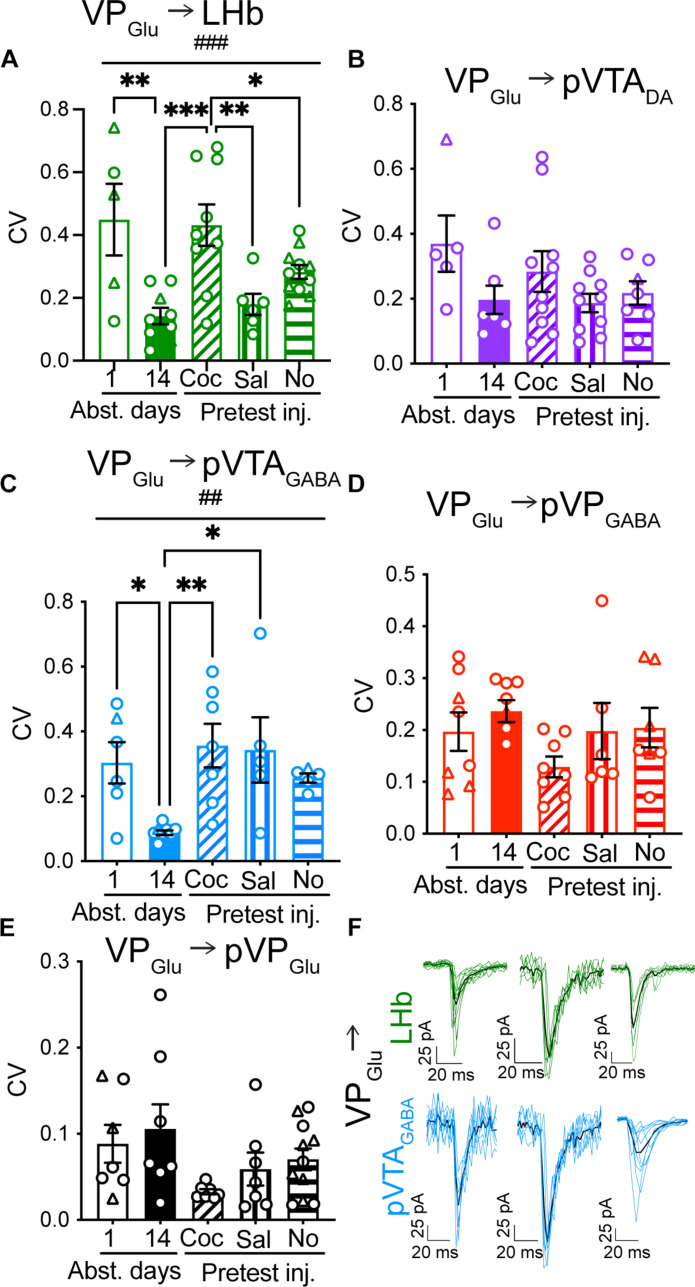
The CV of VP_Glu_ → LHb and VP_Glu_ → pVTA_GABA_ synaptic currents tracks abstinence from and reexposure to cocaine. (A to E) Circles, cells taken from females; triangles, cells taken from males. (**A** and **C**) The CV of the VP_Glu_ projections to the LHb (A) and the pVTA_GABA_ (C) changed across the different stages of the CPP protocol [one-way ANOVA, VP_Glu_ synapses on LHb—###, *F*_(4,34)_ = 6.55, *P* < 0.001; pVTA_GABA_—##, *F*_(4,*2*9)_ = 4.85, *P* = 0.005]. Fourteen days of abstinence after cocaine CPP decreased the CV of both VP_Glu_ → LHb (from 0.45 ± 0.26 to 0.15 ± 0.09; one-way ANOVA, Dunnett’s multiple comparisons test, **P = 0.003) and VP_Glu_ → pVTA_GABA_ (from 0.30 ± 0.16 to 0.09 ± 0.02; one-way ANOVA, Dunnett’s multiple comparisons test, *P = 0.02) synapses, compared to 1 day of abstinence. Reexposure to cocaine after prolonged abstinence significantly increased the CV in both synapses (one-way ANOVA, Dunnett’s multiple comparisons test, ***P = 0.001 for VP_Glu_ → LHb and **P = 0.003 for VP_Glu_ → pVTA_GABA_). The VP_Glu_ → pVTA_GABA_ pathway showed an increase in CV also with a pretest saline injection (one-way ANOVA, Dunnett’s multiple comparisons test, *P* = 0.01) compared to 14 days of abstinence. (**B** and **D**) Cocaine abstinence or reexposure did not affect the CV of the VP_Glu_ synapses on pVTA_DA_, pVP_GABA_, or pVP_Glu_ neurons. (**E**) Reexposure to cocaine after 14 days of abstinence decreased the CV in the VP_Glu_ → pVP_Glu_ synapse (*, one-way ANOVA, Dunnett’s multiple comparisons test, *P* = 0.04). (**F**) Representative traces of currents recorded in VP_Glu_ → LHB and VP_Glu_ → pVTA_GABA_ synapses after 1 and 14 days of abstinence and after reexposure to cocaine. Number of cells ranged between 5 and 11 in all experiments.

To detect changes in the probability of release, we applied two consecutive optogenetic stimulations of the VP_Glu_ terminals while recording from postsynaptic neurons and used two complementary measurements—the paired-pulse ratio (PPR) (Fig. 3) and the coefficient of variation (CV) of evoked postsynaptic current amplitude (Fig. 4) (see Methods). Both measures are considered to be inversely correlated with the probability of release—the higher the probability of release, the lower the PPR and the CV (*19*–*21*).

vGluT2-Cre mice were injected with AAV-DIO-ChR2 into the VP and then underwent cocaine CPP, abstinence, and reexposure to cocaine as also done for the measurement of the A/N ratio (Fig. 2, A and B). A one-way ANOVA test on each projection revealed that only the aversive VP_Glu_ → LHb and VP_Glu_ → pVTA_GABA_ synapses showed a main cocaine condition effect in both the PPR and CV (Figs. 3 and 4; VP_Glu_ → LHb: *F*_(4,35)_ = 4.85, *P* = 0.003 and *F*_(4,35)_ = 7.13, *P* < 0.001 for PPR and CV, respectively; VP_Glu_ → pVTA_GABA_: *F*_(4,29)_ = 4.22, *P* = 0.008 and *F*_(4,29)_ = 4.85, *P* = 0.005 for PPR and CV, respectively; see tables S2 and S3 for full statistical information). Examination within each projection revealed that, like the changes in A/N, the VP_Glu_ → LHb synapse showed presynaptic changes driven by prolonged abstinence or reexposure to cocaine. Prolonged abstinence from cocaine caused a decrease of 33% in the PPR (Fig. 3, A and F; did not reach significance, *P* = 0.26) and a decrease of 68% in the CV (Fig. 4, A and F, *P* = 0.002) in this synapse, suggesting that prolonged abstinence increases the probability of release at the VP_Glu_ → LHb synapse. A cocaine challenge + a CPP test after 14 days of abstinence, but not the mere reintroduction to the CPP box with or without a pretest saline injection, restored the acute abstinence values of both PPR (Fig. 3, A and F; *P* = 0.001 compared to 14 days of abstinence) and CV (Fig. 4, A and F; *P* < 0.001 compared to 14 days of abstinence). Note that the PPR and CV values after a saline injection were significantly different from those after a cocaine injection, supporting the hypothesis that it is the reexposure to cocaine that drives the presynaptic depression in the VP_Glu_ → LHb synapse. Thus, the data suggest that prolonged abstinence from cocaine potentiates and reexposure to cocaine after prolonged abstinence restore preabstinence synaptic release at the VP_Glu_ → LHb through both presynaptic and postsynaptic mechanisms. In contrast to the A/N, the presynaptic values in the VP_Glu_ → LHb synapse did not correlate with the CPP score (fig. S7).

The VP_Glu_ → pVTA_GABA_ synapse showed presynaptic plasticity like that seen in the VP_Glu_ → LHb synapse. Prolonged abstinence from cocaine decreased the PPR by 48% (Fig. 3, C and F; *P* = 0.08) and the CV by 71% (Fig. 4, C and F; *P* = 0.014) compared to the acute abstinence group. Reexposure to cocaine and the CPP test increased both the PPR (2.4-fold, *P* = 0.003) and the CV (4-fold, *P* = 0.003), thus restoring the 1-day-abstinence values. Unlike the VP_Glu_ → LHb synapse, a pretest saline injection increased both the CV (fourfold, *P* = 0.01) and the PPR (twofold, *P* = 0.04, not significant after correcting for multiple comparisons) compared to the prolonged abstinence group (Figs. 3C and 4C). The mere reintroduction to the CPP box had an intermediate effect on the PPR and CV, increasing their values but not as much as a pretest injection did. These data may indicate that the VP_Glu_ → pVTA_GABA_ synapse may encode information about the cues related to the CPP test (the box itself, the injection as a predictor of cocaine) and not only about the pharmacological effect of cocaine. Moreover, both PPR and CV values in this synapse were inversely correlated with the CPP score across all conditions (fig. S7), suggesting that regardless of the presence of cocaine or specific cues, mice that showed higher preference also showed increased probability of release in the VP_Glu_ → pVTA_GABA_ synapse.

The VP_Glu_ → pVP_Glu_ synapse, as the VP_Glu_ → LHb synapse, increased the PPR upon to reexposure to cocaine after prolonged abstinence (Fig. 3E; from 0.74 ± 0.3 to 1.12 ± 0.3, *P* = 0.01). However, reexposure to cocaine did not increase CV but decreased it in this synapse, although this did not reach significance after correcting for multiple comparisons (Fig. 4E; *P* = 0.04).

The VP_Glu_ synapses on the “reward-related” targets, the pVTA_DA_ and pVP_GABA_ neurons, did not show any changes in the PPR (Fig. 3) or CV (Fig. 4) induced either by abstinence from or reexposure to cocaine. The PPR and CV in the VP_Glu_ → pVP_GABA_ did correlate inversely with the CPP score, showing that a higher CPP score is linked to an increased probability of release in this synapse (fig. S7).

Together, our results highlight the VP_Glu_ projections to LHb and pVTA_GABA_ neurons, as well as the intra-VP connections between VP_Glu_ neurons, all known to induce aversion, as most relevant to abstinence from and reexposure to cocaine and the CPP test after prolonged abstinence. In general, prolonged abstinence potentiates, while reexposure to cocaine depresses the VP_Glu_ output to aversion-related targets. These changes occur both presynaptically and postsynaptically in the LHb and only presynaptically in VP_Glu_ → pVTA_GABA_ and VP_Glu_ → pVP_Glu_ synapses.

### VP_Glu_ → LHb and VP_Glu_ → pVTA_GABA_ show the strongest cocaine-induced plasticity

Our data so far limited the plasticity observed to the postsynaptic (Fig. 2) or presynaptic (Figs. 3 and 4) compartments. As the function of a synapse is affected by both compartments, we wanted to visualize the overall change each VP_Glu_ synapse undergoes and identify which VP_Glu_ projections are the most sensitive to prolonged abstinence from cocaine or reexposure to it after prolonged abstinence.

**Fig. 5.**
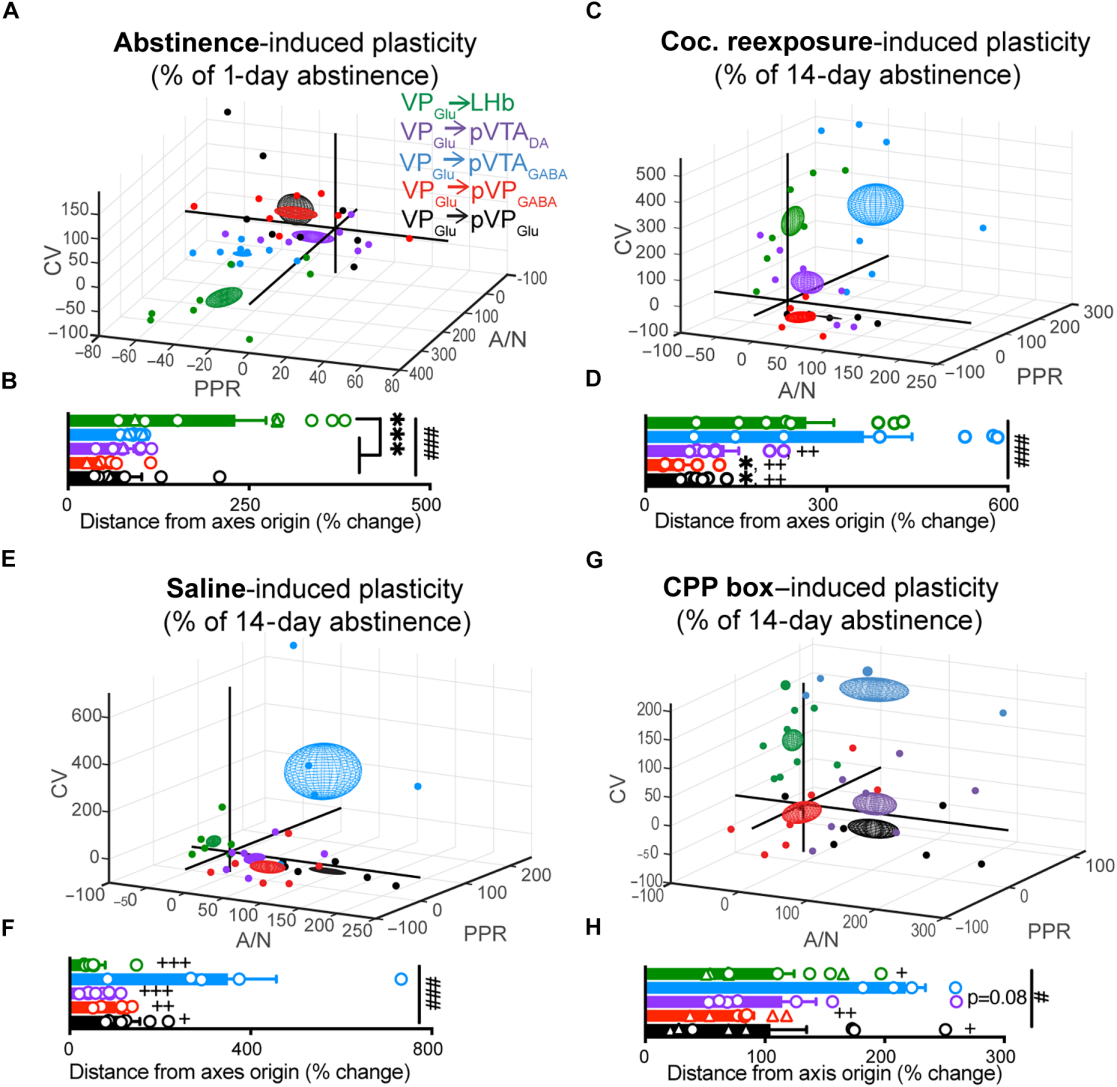
**VP_Glu_** → LHb and VP_Glu_ → pVTA_GABA_ show the strongest plasticity in response to abstinence from and reexposure to cocaine. Circles, cells taken from females; triangles, cells taken from males. For each condition, we provide a three-dimensional graph representing the change in A/N (*x* axis), CV (*y* axis), and PPR (*z* axis) in single cells induced by the respective condition (spheres represent mean change ± SEM in all three parameters for a specific projection), accompanied by a between-projection comparison of the average Euclidean distances from the axis origin. (**A** and **B**) Overall synaptic plasticity driven by prolonged abstinence was different between projections (###, one-way ANOVA, *F*_(4,36)_ = 6.46, *P* = 0.0005). The VP_Glu_ → LHb (219.2, −34.1, and −71.5) stands out as being the most distant from (0,0,0) (***, Tukey’s multiple comparisons test, *P* < 0.001 for VP_Glu_ → LHb compared to each other projection). The VP_Glu_ → pVTA_GABA_ (4.1, −48.4, and −69.8) shows only presynaptic changes in this conditions. (**C** and **D**) Overall synaptic plasticity driven by reexposure to cocaine after prolonged abstinence was different between projections [###, one-way ANOVA, *F*_(4,29)_ = 6.46, *P* = 0.0003]. The VP_Glu_ → LHb (48.8, 116.1, and 201.8) and VP_Glu_ → pVTA_GABA_ (55.0, 141.2, and 306.2) projections show the strongest plasticity [Tukey’s multiple comparisons test, *P* < 0.05 compared to VP_Glu_ → LHb (*), *P* < 0.01 compared to VP_Glu_ → pVTA_GABA_ (++)]. (**E** and **F**) Overall synaptic plasticity driven by reexposure to the CPP box preceded by a saline injection was different between projections [###, one-way ANOVA, *F*_(4,26)_ = 7.60, *P* = 0.0003]. This was driven by the strong plasticity shown only in the VP_Glu_ → pVTA_GABA_ (61.9, 103.4, and 291.2) projection (Tukey’s multiple comparisons test; +/++/+++, *P* < 0.05/*P* < 0.01/*P* < 0.001 compared to VP_Glu_ → pVTA_GABA_). (**G** and **H**) Overall synaptic plasticity driven by reexposure to the CPP box alone was different between projections [#, one-way ANOVA, *F*_(4,31)_ = 3.49, *P* = 0.018]. This was driven by the strong plasticity shown only in the VP_Glu_ → pVTA_GABA_ (58.3, 48.00, and 183.5) projection (Tukey’s multiple comparisons test, all columns compared to VP_Glu_ → pVTA_GABA_; +/++, *P* < 0.05/*P* < 0.01).

To achieve this, we calculated for each neuron the percentage of change in each parameter compared to the preceding condition (i.e., we compared the values after 14 days of abstinence to the average after 1 day of abstinence and the values after the CPP test with cocaine/saline/no pretest injection to the average at 14 days of abstinence). We then plotted neurons on a three-axis graph, each axis representing the change in one of the parameters, with a sphere representing the overall mean ± SEM change for each projection (Fig. 5). We also calculated for each data point its Euclidean distance from the axes origin to evaluate the overall change in each synapse.

**Fig. 6.**
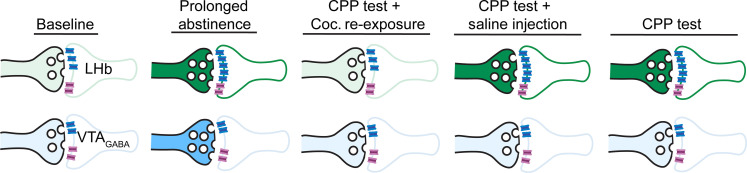
A graphical summary of the overall synaptic plasticity occurring in VP_Glu_ → LHb and VP_Glu_ → pVTA_GABA_ synapses. The number of vesicles represents presynaptic strength, and the number of blue (AMPA) receptors represents postsynaptic strength. Pink receptors, NMDA receptors. Color intensity represents overall synaptic strength. The VP_Glu_ → LHb synapse shows pre- and postsynaptic potentiation after abstinence and depression after reexposure to cocaine. VP_Glu_ → pVTA_GABA_ synapses show similar changes but only on the presynaptic side, as well as presynaptic plasticity when reexposed to cocaine cues, such as the CPP box alone or with a preceding saline injection.

Examination of the overall synaptic changes in each projection highlights the VP_Glu_ → LHb and VP_Glu_ → pVTA_GABA_ as being the most sensitive to prolonged abstinence and reexposure to cocaine and cocaine cues after prolonged abstinence. In particular, the VP_Glu_ → LHb is most sensitive to abstinence from or reexposure to cocaine (Fig. 5, A to D), while the VP_Glu_ → pVTA_GABA_ synapse is highly sensitive to reexposure to cocaine or cocaine cues after abstinence (Fig. 5, C to H). In contrast, the other projections show minor plasticity in these conditions (Fig. 5). Note that exposure to the cues predicting cocaine, the CPP box alone, or with a saline pretest injection caused plasticity only in the VP_Glu_ → pVTA_GABA_ projection, while all other projections were not affected as much (Fig. 5). The overall synaptic changes in each VP_Glu_ synapse driven by each of the conditions tested here are summarized in Fig. 6 and fig. S8.

### Inhibiting VP_Glu_ → LHb but not VP_Glu_ → VTA projection enhances cocaine CPP

**Fig. 7.**
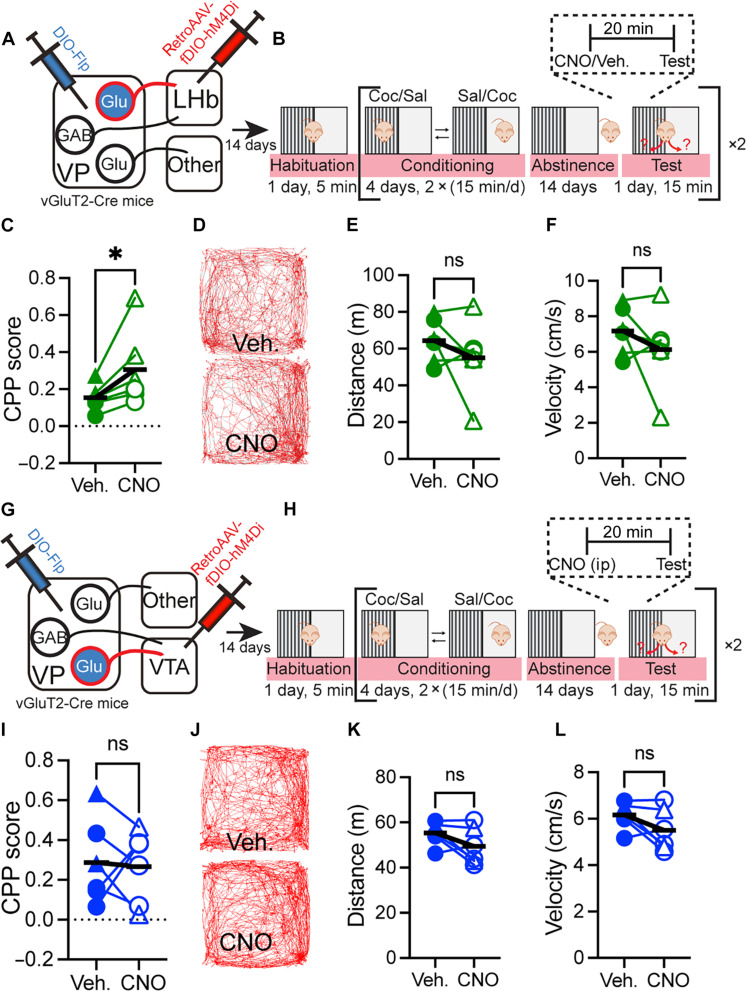
Inhibition of the VP_Glu_ → LHb but not VP_Glu_ → VTA projection enhances cocaine CPP. Circles, females; triangles, males (A to F) VP_Glu_ → LHb. (**A**) We injected a retrograde virus expressing hM4Di in a Flp-dependent manner (retroAAV-fDIO-hM4Di-mCherry) into the LHb and an AAV expressing Flp in a cre-dependent manner (DIO-Flp) into the VP of vGluT2-Cre mice. Thus, only VP_Glu_ → LHb neurons expressed Gi-DREADDs. (**B**) Fourteen days after microinjections, mice went through the cocaine CPP protocol as in Fig. 1A. CNO (3 mg/kg, ip) or vehicle were injected 20 min before the CPP test. Each mouse went through two tests and was injected once with CNO and once with vehicle. Order of CNO/vehicle injections was counterbalanced. (**C**) Inhibition of VP_Glu_ → LHb neurons increased the cocaine CPP score from 0.15 ± 0.07 to 0.31 ± 0.2 (*, paired *t* test; *t*_5_ = 2.61, *P* = 0.031). (**D**) Trajectory movement maps of the same mouse injected with vehicle (top) or CNO (bottom). (**E** and **F**) Inhibiting the VP_Glu_ → LHb neurons did not affect the distance covered by the mice (E) (64.4 ± 12.1 m and 55.1 ± 20.0 m with saline and CNO injections, respectively) or the velocity of movement (F) (7.17 ± 1.36 cm/s and 6.25 ± 2.22 cm/s with saline and CNO injections, respectively). (**G** to **L**) VP_Glu_ → VTA_GABA_. (G and H) Same as in (A) and (B), but retroAAV-fDI-hM4Di-mCherry was injected in the VTA. Thus, only VP_Glu_ → VTA neurons expressed Gi-DREADDs and were inhibited by a CNO injection 20 min before the CPP test (vehicle injected in control trial). (I) Inhibiting the VP_Glu_ → VTA pathway did not generate an overall change in cocaine CPP (CPP score was 0.29 ± 0.2 and 0.27 ± 0.2 with saline and CNO injections, respectively; paired *t* test; *t*_5_ = 0.21, *P* = 0.84). (J) Trajectory movement maps of the same mouse injected with vehicle (top) or CNO (bottom). (K and L) Inhibiting the VP_Glu_ → VTA neurons did not affect the distance covered by the mice (K) (55.4 ± 5.1 m and 49.4 ± 8.2 m with saline and CNO). ns, not significant.

To causally examine whether the VP_Glu_ → LHb projection is the dominant VP_Glu_ projection in modulating CPP, we examined how inhibition of the VP_Glu_ → LHb and VP_Glu_ → VTA projections affect cocaine CPP after abstinence. We injected in vGluT2-Cre mice a virus expressing Flp recombinase in a Cre-dependent manner (AAV-DIO-Flp) into the VP and a retrograde virus expressing Gi-DREADDs in a Flp-dependent manner (retroAAV-fDIO-hM4Di-mCherry) into either the LHb (Fig. 7A and fig. S1) or the VTA (Fig. 7G and fig. S1). We then repeated the cocaine CPP protocol used above (Fig. 1), applying CNO or vehicle 20 min before the beginning of the CPP test (Fig. 7, B and H). As above, each mouse went through two CPP tests separated by reconditioning and abstinence (order of CNO or vehicle injection was counterbalanced). Our data show that inhibiting the VP_Glu_ → LHb projection (Fig. 7, C and D), but not the VP_Glu_ → VTA projection (Fig. 7, I and J), recapitulates the enhancing effect on cocaine CPP we observed when inhibiting the general population of VP_Glu_ neurons (Fig. 1, C and D). While inhibiting the VP_Glu_ → VTA did not change on average cocaine CPP after abstinence (Fig. 7I; CPP score 0.28 ± 0.2 in control, 0.26 ± 0.18 with VP_Glu_ → VTA inhibited), inhibition of the VP_Glu_ → LHb projection doubled the preference for cocaine from 0.15 ± 07 in the control group to 0.31 ± 0.2 (Fig. 7D; see full statistical data in table S1). The differential effect of inhibiting these two projections may be related to our finding that when expressing the DREADDs virus in the VP_Glu_ → LHb projection, we did not observe labeled fibers in the VTA, and when expressing it in the VP_Glu_ → VTA projection, we did not observe labeled fibers in the LHb (fig. S1). Thus, these projections may originate in separate VP_Glu_ neurons. Note that although inhibition of the VP_Glu_ → VTA did not affect CPP on average, it did change CPP in most mice albeit in different directions. This implies that the effect of the VP_Glu_ → VTA projection on CPP may be more nuanced and depend on factors not controlled for here.

## DISCUSSION

The activity level of VP_Glu_ neurons was recently linked to aversion, drug use, and withdrawal. However, there is no knowledge of drug-induced long-term synaptic adaptations in VP_Glu_ neurons after drug exposure and abstinence. In this study, we provide evidence for synaptic plasticity induced by abstinence and reexposure to cocaine or cocaine cues in synapses VP_Glu_ neurons make on LHb and pVTA_GABA_, but not pVTA_DA_ or VP neurons. We show that inhibition of VP_Glu_ neurons (Fig. 1) and specifically their projection to the LHb (Fig. 7) are rewarding and promote cocaine reward. We further show that abstinence from and reexposure to cocaine or cocaine cues are encoded in the synapses that VP_Glu_ neurons make on the LHb and pVTA_GABA_ neurons. Thus, abstinence from cocaine potentiates these synapses, while reexposure to cocaine suppresses them. Synapses that VP_Glu_ neurons make on reward-related targets (pVP_GABA_ and pVTA_DA_ neurons), or on each other, remain largely unaffected. The data point to an “aversive network” activated by VP_Glu_ neurons to encode the progress of cocaine exposure, abstinence, and reexposure.

### VP_Glu_ → VTA_GABA_ as a pathway involved in abstinence from drugs

Recent research has highlighted the VP_Glu_ → LHb pathway as the main output of VP_Glu_ neurons that exerts aversion (*11*). VP_Glu_ neurons make strong synapses on LHb neurons (*17*), and we show here that VP_Glu_ → LHb synapses potentiate during abstinence from cocaine and depress when reexposed to cocaine and that their inhibition is rewarding and elevates cocaine preference. Similar changes were recently shown also for heroin use—the activity of VP_Glu_ → LHb neurons increases during withdrawal from heroin and decreases upon reinstatement of heroin seeking (*18*).

In this work, we also highlight the importance of the VP_Glu_ → VTA_GABA_ projection in the process of cocaine CPP, abstinence, and reintroduction to cocaine and cocaine cues after prolonged abstinence. Like the VP_Glu_ → LHb pathway, but in contrast to the VP_Glu_ input to neighboring VTA_DA_ neurons, the VP_Glu_ → pVTA_GABA_ synapse potentiates after prolonged abstinence and depresses upon subsequent reexposure to cocaine. Nevertheless, the VP_Glu_ → LHb and VP_Glu_ → pVTA_GABA_ projections show some differences. First, while the plasticity in the VP_Glu_ → LHb occurs on both presynaptic and postsynaptic membranes, the plasticity in the VP_Glu_ → pVTA_GABA_ is only presynaptic, occurring only in the VP_Glu_ terminals. Second, abstinence from cocaine induces more dominant plasticity in the VP_Glu_ → LHb pathway. Last, reexposure to the CPP box will only cause plasticity in the VP_Glu_ → LHb synapse if cocaine was injected before the test. These differences in plasticity suggest that the VP_Glu_ → LHb and VP_Glu_ → pVTA_GABA_ may encode different types of information and have different behavioral roles.

### Behavioral roles of VP_Glu_ → LHb versus VP_Glu_ → VTA_GABA_ synapses

Inhibiting the VP_Glu_ neurons increased cocaine CPP (Fig. 1). This was replicated by inhibiting specifically VP_Glu_ → LHb neurons but not by inhibiting VP_Glu_ → VTA neurons (Fig. 7). If VP_Glu_ → LHb synapses encode the negative emotions induced by prolonged abstinence, and if abstinence-induced negative emotions are involved in driving cocaine preference, then inhibiting VP_Glu_ → LHb synapses would have been expected to diminish cocaine motivation. However, the fact that inhibiting the VP_Glu_ → LHb pathway increased cocaine CPP may reflect a priming effect of this manipulation. The LHb is known to decrease dopamine release (*22*, *23*), and as inhibiting the VP_Glu_ input would be expected to decrease LHb activity, it is expected to increase dopamine release. That may be interpreted by the mouse as either a suppression of negative emotions (e.g., “disappointment” of not feeling cocaine-induced reward during the test) or as being rewarding, thereby strengthening cocaine memories, akin to a cocaine priming injection, which is known to increase cocaine preference (*24*, *25*).

Inhibiting the VP_Glu_ → VTA pathway did not change the average cocaine CPP (Fig. 7). It did influence the behavior in most individual mice, but this influence was not consistent. It is thus possible that VP_Glu_ inputs to the VTA have several, possibly opposing roles depending on the neurons they innervate. As VP_Glu_ neurons innervate both VTA_GABA_ and VTA_DA_ neurons (*15*), these two inputs are likely to have opposite effects on dopamine release and thus also on behavior. Our data show that the VP_Glu_ → pVTA_GABA_ and VP_Glu_ → pVTA_DA_ synapses change differentially during abstinence from and reexposure to cocaine (Figs. 2 to 5). In addition, similar diversity in the effects of VP_Glu_ inputs to the VTA was reported recently as optogenetic activation of VP_Glu_ terminals in the VTA generated diverse dopamine release patterns in the nucleus accumbens (*18*). Unfortunately, it is still not possible technologically to manipulate the activity of specific synapses. Thus, although we predict that inhibiting VP_Glu_ → VTA_GABA_ synapses would be rewarding and possibly enhance cocaine CPP, while inhibiting VP_Glu_ → VTA_DA_ would be aversive and decrease cocaine CPP, we cannot test this hypothesis experimentally with current available tools.

Nevertheless, a possible hint for a more specific behavioral role of the VP_Glu_ → pVTA_GABA_ neurons may come from the different plasticity patterns it shows compared to the VP_Glu_ → LHb pathway when reintroduced to the CPP box after prolonged abstinence. While the VP_Glu_ → LHb synapse seems to respond mainly to the reexposure to cocaine (especially presynaptically), the VP_Glu_ → pVTA_GABA_ synapse responds also to the reintroduction to the CPP box with or without a preceding saline injection (Figs. 2 to 6). Thus, the VP_Glu_ → LHb synapse may encode the fluctuations in the animal’s emotional state (reward versus aversion), while the VP_Glu_ → pVTA_GABA_ may be more directed at encoding reward-predicting cues. VTA_GABA_ neurons are involved in encoding reward-predictive cues (*26*–*28*), and our data suggest that their input from the VP_Glu_ neurons may take part in this role.

Last, as much as our data highlight the VP_Glu_ inputs to the LHb and pVTA_GABA_ as having unique roles on behavior, they may as well be part of a general excitatory drive on these targets that modulates aversion and reward. Thus, previous studies show that excitatory inputs from the hypothalamus and entopeduncular nucleus to the LHb (*29*–*31*) regulate aversion, and the VTA_GABA_ neurons receive diverse excitatory inputs that were speculated, although not directly tested, to reduce reward and enhance aversion (*26*, *32*). Further research would be needed to test whether the VP_Glu_ input to the LHb or VTA_GABA_ neurons carries unique behavioral information or is one of many that simply drives the activation of these targets.

### VP_Glu_, LHb, and VTA_GABA_ as an aversive network in abstinence

The LHb (*31*, *33*–*36*), VTA_GABA_ (*37*–*41*), and VP_Glu_ neurons (*14*, *17*, *18*) all generate aversion, are activated by aversive stimuli, and are involved in a variety of psychiatric disorders. The LHb and VTA_GABA_ neurons inhibit dopamine release. VP_Glu_ neurons were suggested to depress dopamine release as well, although data show a heterogeneous effect on VTA_DA_ activity (*14*, *18*). We have shown in a previous study that VP_Glu_ neurons form their strongest synapses on neurons whose activation induces aversion, including LHb, VTA_GABA_, and other VP_Glu_ neurons (*15*), and the activity of these neurons is elevated by aversive stimuli (*10*, *18*). Recent studies demonstrate a role for VP_Glu_ neurons in drug seeking and withdrawal—their activation reduces seeking of cocaine (*13*) and heroin (*16*), and they increase their activity during withdrawal (*16*). As with their effect on aversion, the VP_Glu_ effects on withdrawal are thought to involve the projection to the LHb (*16*).

Our data expand these notions to suggest a synaptic “triangle of aversion” in abstinence from drugs. First, we emphasize VTA_GABA_ neurons as another important target of VP_Glu_ neurons in the context of drug abstinence and exposure. Abstinence from cocaine potentiates this synapse, while reexposure to cocaine after abstinence suppresses this synapse and restores preabstinence values (Figs. 3 to 6). Second, we show that drug- and abstinence-induced changes are embedded in the VP_Glu_ → LHb and VP_Glu_ → pVTA_GABA_ pathways at the synaptic level—both synapses, particularly the VP_Glu_ → LHb synapse, show dynamic synaptic changes as a mouse progresses from drug exposure to abstinence to reexposure.

Given that VTA_GABA_ neurons inhibit dopamine release from VTA_DA_ neurons (*36*–*38*) and that LHb neurons activate VTA_GABA_ neurons to inhibit dopamine release (*39*), our data propose that VP_Glu_ neurons may orchestrate the dynamics of the release of dopamine during abstinence or reexposure to cocaine. Thus, it may cause a decrease in dopamine release after abstinence by activating more strongly LHb and VTA_GABA_ neurons while enhancing dopamine release upon reexposure to cocaine by depressing VP_Glu_ synapses on both targets. Thus, dopamine levels may rise, at least temporarily, to end the negative affective state.

## METHODS

### Animals

We used male and female 8- to 12-week-old vGluT2-IRES-Cre transgenic mice (the Jackson Laboratory, strain #016963) on a C57bl6/J background for the behavioral experiments (Figs. 1 and 6). For the electrophysiological experiments (Figs. 2 to 4), we crossed these mice with Ai9 mice (the Jackson Laboratory, strain #007909) to express tdTomato in glutamatergic neurons, and 8- to 12-week male and female vGluT2-IRES-Cre X Ai9 were used in the experiments. All mice were group-housed (between two and five mice per cage, males and females separately) in a reverse light cycle (lights off at 08:00 a.m.), and experiments were conducted between 09:00 and 19:00. Regular chow and drinking water were available ad libitum. All procedures performed here were approved by the Research Animal Care Committee of the Hebrew University under license numbers MD-15-14405-4 and MD-19-15891-4.

### Viral injections

Viral injections were performed as described in our previous study (*15*). Briefly, we anesthetized mice with isoflurane and fixed them in a stereotaxic frame (Kopf, model 940). In the experiments of Figs. 1 to 5, we drilled bilateral holes in the skull, and 300 nl of virus was microinjected into the VP [coordinates in millimeters relative to bregma: anterior/posterior (A/P), +0.4; medial/lateral (M/L), 1.1; dorsal/ventral (D/V), −5] through a 30 G NanoFil syringe (World Precision Instruments, FL, USA) (100 nl/min; the needle was retracted 5 min after the injection was terminated). The viral constructs were either AAV2-hSyn-DIO-hM4Di-mCherry (Fig. 1) or AAV2-EF1a-DIO-hChR2(H134R)-eYFP (enhanced Yellow Fluorescence Protein) (Figs. 2 to 5) (University of North Carolina Viral Core). In the experiments of Fig. 7, two sets of bilateral holes were drilled—one above the VP and the other above the LHb or VTA. AAV2-DIO-Flp (Addgene, #87306) was injected (300 nl) into the VP, while retroAAV-fDIO-hM4Di-mCherry (Addgene, #154867) was injected together with AAV-hSyn-eYFP to visualize the injection site (300 nl each) into either the LHb (A/P, −1.5; M/L, 0.5; D/V, −3) or the VTA (A/P, −2.1; M/L, 0.31; D/V, −4.6). Sham mice were injected with AAV2-DIO-eYFP (Addgene, #27056) into the VP and retroAAV-fDIO-hM4Di-mCherry into the LHb or VTA. Mice were allowed to recover for at least 1 week before experiments began. At the end of all experiments, we prepared brain slices to examine the location of the viral injection(s). Mice in which the focus of the viral injection (i.e., the point with the strongest fluorescence) was outside the borders of the injected region determined by the Paxinos mouse brain atlas (*42*), or in which the injection spot was on target, but the fluorescence spread substantially outside the targeted region (approximately more than 20% of the fluorescence, estimated by eye), were not analyzed. Note that while in the behavioral experiments (Figs. 1 and 6) we discarded the whole mouse, in the electrophysiological experiments, we discarded only the relevant hemisphere and still used the other. Overall, two mice were discarded from the behavioral experiments, and five hemispheres from five different mice were discarded from the electrophysiological experiments.

### Cocaine CPP

The cocaine CPP and abstinence protocol we used here is similar to that described in our previous studies (*43*, *44*). Behavioral procedures started at least 1 week after microinjections, when the mice were ~10 weeks old and acclimated to the reverse light cycle. All mice were trained in the unbiased two-chamber cocaine CPP paradigm that provides good estimate of reward preference—a 30 cm–by–30 cm arena was divided by a plastic separating wall in two, each side with different wall patterns and floor texture (Fig. 1B). On the first day, all mice were allowed to explore the arena freely. Then, experimental mice went through two conditioning sessions per day (5 hours apart), in which they received either cocaine [in the paired side, 15 mg/kg, intraperitoneally (ip)] or saline (in the unpaired side). The order of injections and the side of the box that was paired with cocaine were balanced between groups. After four conditioning days (eight sessions), mice were left in their home cages for 14 days before being tested for preference of the cocaine-paired side (the separating wall was removed during the test).

In the electrophysiological experiments (Figs. 2 to 5), mice were tested by either putting them in the box without treatment, immediately after a priming injection of cocaine (15 mg/kg ip), or after an intraperitoneal saline injection. Mice were taken for electrophysiological recordings 15 min after the beginning of the test. Two additional groups of mice were taken for recordings 1 or 14 days after the end of the last conditioning session. These mice were taken for recordings directly from their home cages at approximately the same time of the day as all other mice.

For the behavioral experiments in Fig. 1, mice were injected with either CNO (3 mg/kg, ip) or saline 20 min before the beginning of the test and were then placed in the box without any other pretreatment for 15 min. To allow us to measure the preference for cocaine twice in the same mouse, once with CNO and once with saline, mice underwent the cocaine conditioning protocol followed by the 14-day abstinence once again, 24 hours after the first test. The order of CNO/saline injections was counterbalanced—half the mice received CNO before the first CPP test and half the mice received a saline injection before the first CPP test. Mouse behavior and movement were recorded with cameras and analyzed offline (Ethovision X, Noldus). Preference for the cocaine-paired side was calculated as the ratio between the difference in time spent between the cocaine-paired and unpaired sides and the total time [CPP score = (time in paired zone − time in unpaired zone)/(time in paired zone + time in unpaired zone)]. Using this method, a CPP score of 1 is complete preference, a score of (−1) is complete avoidance, and a CPP score of zero represents indifference.

### Preference for the side paired with VP_Glu_ inhibition

Mice expressing the Gi-DREADD in VP_Glu_ neurons underwent the same protocol described above for cocaine CPP but were injected with CNO (3 mg/kg, ip, 20 min before the session) instead of cocaine during conditioning. After 14 days of abstinence, they were reintroduced to the box without any pretreatment, and their movement was recorded for 15 min.

### Slice preparation for patch-clamp recordings

Slices for patch-clamp recordings were prepared as described in our previous study (*15*). Mice were anesthetized [ketamine HCl (150 mg/kg)] and decapitated, and coronal, sagittal, or horizontal slices (200 m) of the VP, LHb, or VTA were prepared (VT1200S Leica vibratome). Slices were transferred to a vial containing artificial cerebrospinal fluid (aCSF; 126 mM NaCl, 1.4 mM NaH_2_PO_4_, 25 mM NaHCO_3_, 11 mM glucose, 1.2 MgCl_2_, 2.4 mM CaCl_2_, 2.5 mM KCl, 2.0 mM Na-pyruvate, and 0.4 mM ascorbic acid, bubbled with 95% O_2_ and 5% CO_2_) and a mixture of 5 mM kynurenic acid and 50 µM D-2-amino-5-phosphonovaleric acid (D-AP5). Slices were stored at room temperature (22° to 24°C) until recording.

### In vitro whole-cell recording

All recordings were collected at 32°C (TC-344B, Warner Instrument Corporation). Ventral pallidal neurons were visualized with an Olympus BX51WI microscope. Inhibitory synaptic transmission was blocked with picrotoxin (0.1 mM). Multiclamp 700B (Axon Instruments, Union City, CA) was used to record excitatory postsynaptic currents in whole-cell configuration. Glass microelectrodes (1.3 to 2 megohm) were filled with internal solution [128 mM cesium methanesulfonate, 10 mM Hepes potassium, 1 mM EGTA, 1 mM MgCl_2_, 10 mM NaCl, 2.0 mM Mg–adenosine triphosphate, 0.3 mM Na–guanosine triphosphate, and 1 mM QX-314 (pH 7.2 to 7.3), ~280 mOsm]. Data were acquired at 10 kHz and filtered at 2 kHz using AxoGraph X software (AxoGraph Scientific, Sydney). For optogenetic stimulation, we used a 470-nm light-emitting diode light source (Mightex Systems, CA) directed to the slice through the objective. If the optogenetic stimulation triggered a noticeable (amplitude of at least 50 pA) postsynaptic current that started up to 2 ms after the beginning of the optogenetic stimulation and appeared consistently in at least 80% of stimulations, we considered the neuron as receiving VP_Glu_ input and continued recording. Proportions of responding neurons were reported in our previous study (*15*). For recording of the PPR and the CV, we held the neurons at a membrane potential of −70 mV, applied two consecutive optogenetic stimulations (1 ms long), and measured the amplitudes of 20 to 30 evoked currents. The CV was calculated by dividing the standard deviation of the amplitudes of the first evoked currents by the mean amplitude of these currents. The PPR was calculated by dividing the amplitude of the second current by that of the first current. For measuring the AMPA/NMDA ratio, we held neurons at +40 mV for 5 min, then applied an optogenetic stimulation, and recorded the excitatory currents (10 to 20 repetitions). Then, we added the NMDA receptor antagonist D-AP5 (50 μM), allowed it to take action for 3 min, and then applied the optogenetic stimulation again to record the excitatory current that lacked the NMDA component, presumably consisting of only AMPA-mediated currents. Last, we subtracted the AMPA-mediated current from the total current to yield the NMDA-mediated current. The AMPA/NMDA ratio represents the ratio between the peaks of the currents. Recordings started 10 min after membrane rupture and were collected every 20 s. Series resistance (*R*_s_), measured with a −2-mV depolarizing step (10 ms) given with each stimulus, was always monitored online. Recordings with unstable *R*_s_ or when *R*_s_ exceeded 20 megohm were aborted.

### Cell type identification

The identity of neurons in the VP and the VTA was determined by a combination of parameters. pVP_Glu_ neurons were primarily identified by fluorescence of tdTomato. Note that vGluT2 has been shown to be expressed temporarily during development in some cell types that did not express vGluT2 in adulthood (*45*, *46*). Although this was not shown in the VP, we refer to the tdTomato-positive neurons as pVP_Glu_ neurons. The pVP_Glu_ neurons are highly likely to be glutamatergic neurons as the prevalence of these neurons in the VP is about 10% (*47*, *48*), and we have found that in our slices, the labeled neurons in the VP make ~7% of all neurons (fig. S5). Tdtomato-negative neurons were assumed to be pVP_GABA_ neurons based on their reported high prevalence [~90%; (*14*, *15*)], the fact that they do not express tdTomato, and the difference in physiology and morphology from the third most abundant type of VP neurons, cholinergic neurons. Morphologically, the soma diameter of cholinergic neurons is twice bigger than that of noncholinergic neurons (30 μm versus 14 μm, respectively) (*49*), and thus we avoided somas that were in the range of 30 μm. In addition, cholinergic neurons in the VP show a more hyperpolarized membrane potential, around −65 mV (*49*), than noncholinergic neurons (*50*). Thus, we discarded tdTomato-negative neurons with a large soma and a membrane potential more negative than −60 mV. Note that with our tools, we cannot rule out that some neurons may have coexpressed GABA and glutamate, although such neurons have not been reported in the VP to our knowledge.

In the VTA, we used a different set of morphological and physiological parameters to distinguish between pVTA_DA_ and pVTA_GABA_ neurons. Morphologically, we show here (fig. S6), and others have shown before (*51*), that dopaminergic neurons have bigger soma sizes than GABAergic neurons (17.4 ± 2.1 μm and 13.4 ± 1.8 μm, respectively; 30% difference, *P* < 0.0001). Thus, we used the size of the soma as one parameter to distinguish between pVTA_DA_ and pVTA_GABA_ neurons. Also, as we used vGluT2-Cre X Ai9 mice, we excluded neurons expressing td-Tomato. Electrophysiologically, the presence of a hyperpolarization-induced current (*I*_h_) was suggested to be characteristic of dopaminergic neurons (*51*, *52*). Although recent studies show that such currents can be found also in VTA_GABA_ neurons (*53*, *54*) and there are dopamine neurons that do not express *I*_h_ currents (*55*), we applied hyperpolarizatory steps in each cell and found that the neurons we identified as pVTA_DA_ had substantial *I*_h_ currents (48.2 ± 67.9 pA), while the pVTA_GABA_ did not (−0.98 ± 3.61 pA; *P* = 0.003) (fig. S6). Thus, nonglutamatergic neurons with bigger somas that exhibited *I*_h_ currents were considered to be pVTA_DA_, while nonglutamatergic neurons with smaller somas that did not express *I*_h_ were considered pVTA_GABA_ neurons. We cannot rule out that some of the neurons may have coexpressed both GABA and dopamine.

### Data analysis

To calculate the percent change in A/N, PPR, or CV between 14 days and 1 day of abstinence in Fig. 5, we calculated for each cell in the 14-day abstinence group the difference between a recorded parameter (e.g., A/N) and the average value of the same parameter in the same projection in the 1-day abstinence group. We then divided this difference by the average value in the 1-day abstinence group to yield the percent change. Similar logic was used to calculate the percent change driven by a cocaine injection/saline injection/no injection after abstinence, but in these groups, the change was calculated relative to the 14-day abstinence group.

After calculating for each neuron its percent change in all three parameters (overall 134 neurons with all three parameters), we generated for each of the four conditions (the effect of prolonged abstinence and the effects of a cocaine injection, a saline injection or no injection after prolonged abstinence) a three-axis graph (A/N, PPR, and CV) on which we plotted the neurons, color-coded by projection using MATLAB (MathWorks, USA). For each projection in each graph, we also calculated the mean and SEM, which is represented as a sphere on the graph. The Euclidean distance of each data point from the axis origin was calculated as the square root of the sum of square values on each axis.

### Statistical analysis

Statistics were performed using GraphPad Prism 10.0 (GraphPad Software Inc., San Diego, CA). Outliers were identified using the robust regression and outlier removal (ROUT) method (embedded within GraphPad Prism 10) with *Q* = 1 (i.e., aiming for 1% of false identified outliers). Using this method, we identified 12 outliers of 569 values measured in the electrophysiology experiments (there were no outliers in the behavioral experiments). Parametric statistics (paired Student’s *t* test or one-way ANOVA) were used unless stated otherwise. Mouse behavior was analyzed and quantified using Ethovision X (Noldus, the Netherlands). In the electrophysiological experiments (Figs. 2 to 4), we used the Bonferroni correction for multiple comparisons across all tests. This is because we compared three parameters (A/N, PPR, and CV) between the same neurons. Thus, the threshold *P* value for rejection of the null hypothesis is not *P* = 0.05 but *P* = 0.05/3 = 0.0167.

## References

[1] L. Lu, J. W. Grimm, B. T. Hope, Y. Shaham, Incubation of cocaine craving after withdrawal: A review of preclinical data. Neuropharmacology 47, 214–226 (2004). 15464139 10.1016/j.neuropharm.2004.06.027

[2] C. Chavkin, G. F. Koob, Dynorphin, dysphoria, and dependence: The stress of addiction. Neuropsychopharmacology 41, 373–374 (2016). 10.1038/npp.2015.258 PMC4677142 26657953

[3] A. K. Zinsmaier, Y. Dong, Y. H. Huang, Cocaine-induced projection-specific and cell type-specific adaptations in the nucleus accumbens. Mol. Psychiatry 27, 669–686 (2022). 33963288 10.1038/s41380-021-01112-2 PMC8691189

[4] Y. Dong, J. R. Taylor, M. E. Wolf, Y. Shaham, Circuit and synaptic plasticity mechanisms of drug relapse. J. Neurosci. 37, 10867–10876 (2017). 29118216 10.1523/JNEUROSCI.1821-17.2017 PMC5678019

[5] V. Pascoli, J. Terrier, J. Espallergues, E. Valjent, E. C. O’Connor, C. Lüscher, Contrasting forms of cocaine-evoked plasticity control components of relapse. Nature 509, 459–464 (2014). 24848058 10.1038/nature13257

[6] L. Wills, P. J. Kenny, Addiction-related neuroadaptations following chronic nicotine exposure. J. Neurochem. 157, 1652–1673 (2021). 33742685 10.1111/jnc.15356 PMC13249106

[7] K. L. Conrad, K. Y. Tseng, J. L. Uejima, J. M. Reimers, L.-J. Heng, Y. Shaham, M. Marinelli, M. E. Wolf, Formation of accumbens GluR2-lacking AMPA receptors mediates incubation of cocaine craving. Nature 454, 118–121 (2008). 18500330 10.1038/nature06995 PMC2574981

[8] Y. M. Kupchik, A. A. Prasad, Ventral pallidum cellular and pathway specificity in drug seeking. Neurosci. Biobehav. Rev. 131, 373–386 (2021). 34562544 10.1016/j.neubiorev.2021.09.007

[9] C. Soares-Cunha, J. A. Heinsbroek, Ventral pallidal regulation of motivated behaviors and reinforcement. Front. Neural Circuits. 17, 1086053 (2023). 36817646 10.3389/fncir.2023.1086053 PMC9932340

[10] M. Stephenson-Jones, C. Bravo-Rivera, S. Ahrens, A. Furlan, X. Xiao, C. Fernandes-Henriques, B. Li, Opposing contributions of gabaergic and glutamatergic ventral pallidal neurons to motivational behaviors. Neuron 105, 921–933.e5 (2020). 31948733 10.1016/j.neuron.2019.12.006 PMC8573387

[11] L. Faget, V. Zell, E. Souter, A. McPherson, R. Ressler, N. Gutierrez-Reed, J. H. Yoo, D. Dulcis, T. S. Hnasko, Opponent control of behavioral reinforcement by inhibitory and excitatory projections from the ventral pallidum. Nat. Commun. 9, 849 (2018). 29487284 10.1038/s41467-018-03125-y PMC5829073

[12] D. H. Root, A. T. Fabbricatore, A. P. Pawlak, D. J. Barker, S. Ma, M. O. West, Slow phasic and tonic activity of ventral pallidal neurons during cocaine self-administration. Synapse 66, 106–127 (2012). 21953543 10.1002/syn.20990 PMC4106448

[13] J. A. Heinsbroek, A.-C. Bobadilla, E. Dereschewitz, A. Assali, R. M. Chalhoub, C. W. Cowan, P. W. Kalivas, Opposing regulation of cocaine seeking by glutamate and GABA neurons in the ventral pallidum. Cell Rep. 30, 2018–2027.e3 (2020). 32049028 10.1016/j.celrep.2020.01.023 PMC7045305

[14] J. Tooley, L. Marconi, J. B. Alipio, B. Matikainen-Ankney, P. Georgiou, A. V. Kravitz, M. C. Creed, Glutamatergic ventral pallidal neurons modulate activity of the habenula-tegmental circuitry and constrain reward seeking. Biol. Psychiatry 83, 1012–1023 (2018). 29452828 10.1016/j.biopsych.2018.01.003 PMC5972062

[15] L. A. Levi, K. Inbar, N. Nachshon, N. Bernat, A. Gatterer, D. Inbar, Y. M. Kupchik, Projection-specific potentiation of ventral pallidal glutamatergic outputs after abstinence from cocaine. J. Neurosci. 40, 1276–1285 (2020). 31836662 10.1523/JNEUROSCI.0929-19.2019 PMC7002147

[16] R.-S. Chen, J. Liu, Y.-J. Wang, K. Ning, J.-G. Liu, Z.-Q. Liu, Glutamatergic neurons in ventral pallidum modulate heroin addiction via epithalamic innervation in rats. Acta Pharmacol. Sin. 45, 945–958 (2024). 38326624 10.1038/s41401-024-01229-4 PMC11053033

[17] F. Wang, J. Zhang, Y. Yuan, M. Chen, Z. Gao, S. Zhan, C. Fan, W. Sun, J. Hu, Salience processing by glutamatergic neurons in the ventral pallidum. Sci. Bull. 65, 389–401 (2020). 10.1016/j.scib.2019.11.029 36659230

[18] L. Faget, L. Oriol, W.-C. Lee, V. Zell, C. Sargent, A. Flores, N. G. Hollon, D. Ramanathan, T. S. Hnasko, Ventral pallidum GABA and glutamate neurons drive approach and avoidance through distinct modulation of VTA cell types. Nat. Commun. 15, 4233 (2024). 38762463 10.1038/s41467-024-48340-y PMC11102457

[19] D. S. Faber, H. Korn, Applicability of the coefficient of variation method for analyzing synaptic plasticity. Biophys. J. 60, 1288–1294 (1991). 1684726 10.1016/S0006-3495(91)82162-2 PMC1260182

[20] A. F. Schinder, B. Berninger, M. Poo, Postsynaptic target specificity of neurotrophin-induced presynaptic potentiation. Neuron 25, 151–163 (2000). 10707980 10.1016/s0896-6273(00)80879-x

[21] B. Berninger, A. F. Schinder, M. M. Poo, Synaptic reliability correlates with reduced susceptibility to synaptic potentiation by brain-derived neurotrophic factor. Learn. Mem. 6, 232–242 (1999). 10492005 PMC311306

[22] H. Ji, P. D. Shepard, Lateral habenula stimulation inhibits rat midbrain dopamine neurons through a GABA_A_ receptor-mediated mechanism. J. Neurosci. 27, 6923–6930 (2007). 17596440 10.1523/JNEUROSCI.0958-07.2007 PMC6672239

[23] P. L. Brown, H. Palacorolla, D. Brady, K. Riegger, G. I. Elmer, P. D. Shepard, Habenula-induced inhibition of midbrain dopamine neurons is diminished by lesions of the rostromedial tegmental nucleus. J. Neurosci. 37, 217–225 (2017). 28053043 10.1523/JNEUROSCI.1353-16.2016 PMC5214632

[24] D. Mueller, J. Stewart, Cocaine-induced conditioned place preference: Reinstatement by priming injections of cocaine after extinction. Behav. Brain Res. 115, 39–47 (2000). 10996406 10.1016/s0166-4328(00)00239-4

[25] Y. Itzhak, J. L. Martin, Cocaine-induced conditioned place preference in mice: Induction, extinction and reinstatement by related psychostimulants. Neuropsychopharmacology 26, 130–134 (2002). 11751040 10.1016/S0893-133X(01)00303-7

[26] C. Bouarab, B. Thompson, A. M. Polter, VTA GABA neurons at the interface of stress and reward. Front. Neural Circuits 13, 78 (2019). 31866835 10.3389/fncir.2019.00078 PMC6906177

[27] W.-X. Pan, J. Brown, J. T. Dudman, Neural signals of extinction in the inhibitory microcircuit of the ventral midbrain. Nat. Neurosci. 16, 71–78 (2013). 23222913 10.1038/nn.3283 PMC3563090

[28] J. Y. Cohen, S. Haesler, L. Vong, B. B. Lowell, N. Uchida, Neuron-type-specific signals for reward and punishment in the ventral tegmental area. Nature 482, 85–88 (2012). 22258508 10.1038/nature10754 PMC3271183

[29] S. J. Shabel, C. D. Proulx, A. Trias, R. T. Murphy, R. Malinow, Input to the lateral habenula from the basal ganglia is excitatory, aversive, and suppressed by serotonin. Neuron 74, 475–481 (2012). 22578499 10.1016/j.neuron.2012.02.037 PMC3471532

[30] I. Lazaridis, O. Tzortzi, M. Weglage, A. Märtin, Y. Xuan, M. Parent, Y. Johansson, J. Fuzik, D. Fürth, L. E. Fenno, C. Ramakrishnan, G. Silberberg, K. Deisseroth, M. Carlén, K. Meletis, A hypothalamus-habenula circuit controls aversion. Mol. Psychiatry 24, 1351–1368 (2019). 30755721 10.1038/s41380-019-0369-5 PMC6756229

[31] S. Lecca, F. J. Meye, M. Trusel, A. Tchenio, J. Harris, M. K. Schwarz, D. Burdakov, F. Georges, M. Mameli, Aversive stimuli drive hypothalamus-to-habenula excitation to promote escape behavior. eLife 6, e30697 (2017). 28871962 10.7554/eLife.30697 PMC5606847

[32] L. Faget, F. Osakada, J. Duan, R. Ressler, A. B. Johnson, J. A. Proudfoot, J. H. Yoo, E. M. Callaway, T. S. Hnasko, afferent inputs to neurotransmitter-defined cell types in the ventral tegmental area. Cell Rep. 15, 2796–2808 (2016). 27292633 10.1016/j.celrep.2016.05.057 PMC4917450

[33] A. M. Stamatakis, G. D. Stuber, Activation of lateral habenula inputs to the ventral midbrain promotes behavioral avoidance. Nat. Neurosci. 15, 1105–1107 (2012). 22729176 10.1038/nn.3145 PMC3411914

[34] B. Li, J. Piriz, M. Mirrione, C. Chung, C. D. Proulx, D. Schulz, F. Henn, R. Malinow, Synaptic potentiation onto habenula neurons in the learned helplessness model of depression. Nature 470, 535–539 (2011). 21350486 10.1038/nature09742 PMC3285101

[35] M. Maroteaux, M. Mameli, Cocaine evokes projection-specific synaptic plasticity of lateral habenula neurons. J. Neurosci. 32, 12641–12646 (2012). 22956853 10.1523/JNEUROSCI.2405-12.2012 PMC6621263

[36] W. Zuo, L. Chen, L. Wang, J.-H. Ye, Cocaine facilitates glutamatergic transmission and activates lateral habenular neurons. Neuropharmacology 70, 180–189 (2013). 23347950 10.1016/j.neuropharm.2013.01.008 PMC3644336

[37] E. H. Mitten, A. Souders, E. Marron Fernandez de Velasco, K. Wickman, Stress-induced anxiety-related behavior in mice is driven by enhanced excitability of ventral tegmental area GABA neurons. Front. Behav. Neurosci. 18, 1425607 (2024). 39086371 10.3389/fnbeh.2024.1425607 PMC11288924

[38] D. C. Lowes, L. A. Chamberlin, L. N. Kretsge, E. S. Holt, A. I. Abbas, A. J. Park, L. Yusufova, Z. H. Bretton, A. Firdous, A. G. Enikolopov, J. A. Gordon, A. Z. Harris, Ventral tegmental area GABA neurons mediate stress-induced blunted reward-seeking in mice. Nat. Commun. 12, 3539 (2021). 34112787 10.1038/s41467-021-23906-2 PMC8192742

[39] D. H. Root, D. J. Barker, D. J. Estrin, J. A. Miranda-Barrientos, B. Liu, S. Zhang, H.-L. Wang, F. Vautier, C. Ramakrishnan, Y. S. Kim, L. Fenno, K. Deisseroth, M. Morales, Distinct signaling by ventral tegmental area glutamate, GABA, and combinatorial glutamate-GABA neurons in motivated behavior. Cell Rep. 32, 108094 (2020). 32877676 10.1016/j.celrep.2020.108094 PMC7556367

[40] R. van Zessen, J. L. Phillips, E. A. Budygin, G. D. Stuber, Activation of VTA GABA neurons disrupts reward consumption. Neuron 73, 1184–1194 (2012). 22445345 10.1016/j.neuron.2012.02.016 PMC3314244

[41] K. R. Tan, C. Yvon, M. Turiault, J. J. Mirzabekov, J. Doehner, G. Labouèbe, K. Deisseroth, K. M. Tye, C. Lüscher, GABA neurons of the VTA drive conditioned place aversion. Neuron 73, 1173–1183 (2012). 22445344 10.1016/j.neuron.2012.02.015 PMC6690362

[42] G. Paxinos, K. B. J. Franklin, *The Mouse Brain in Stereotaxic Coordinates* (Academic Press, ed. 2, 2001).

[43] K. Inbar, L. A. Levi, Y. M. Kupchik, Cocaine induces input and cell-type-specific synaptic plasticity in ventral pallidum-projecting nucleus accumbens medium spiny neurons. Neuropsychopharmacology 47, 1461–1472 (2022). 35121830 10.1038/s41386-022-01285-6 PMC9205871

[44] N. Bernat, R. R. Campbell, H. Nam, M. Basu, T. Odesser, G. Elyasaf, M. Engeln, R. Chandra, S. Golden, S. Ament, M. K. Lobo, Y. M. Kupchik, Multimodal interrogation of ventral pallidum projections reveals projection-specific signatures and effects on cocaine reward. J. Neurosci. 44, e1469232024 (2024). 38485256 10.1523/JNEUROSCI.1469-23.2024 PMC11063828

[45] J. A. Mendez, M.-J. Bourque, G. Dal Bo, M. L. Bourdeau, M. Danik, S. Williams, J.-C. Lacaille, L.-E. Trudeau, Developmental and target-dependent regulation of vesicular glutamate transporter expression by dopamine neurons. J. Neurosci. 28, 6309–6318 (2008). 18562601 10.1523/JNEUROSCI.1331-08.2008 PMC6670902

[46] T. Steinkellner, V. Zell, Z. J. Farino, M. S. Sonders, M. Villeneuve, R. J. Freyberg, S. Przedborski, W. Lu, Z. Freyberg, T. S. Hnasko, Role for VGLUT2 in selective vulnerability of midbrain dopamine neurons. J. Clin. Invest. 128, 774–788 (2018). 29337309 10.1172/JCI95795 PMC5785252

[47] L. Yang, L. Z. Fang, M. R. Lynch, C. S. Xu, H. J. Hahm, Y. Zhang, M. R. Heitmeier, V. D. Costa, V. K. Samineni, M. C. Creed, Transcriptomic landscape of mammalian ventral pallidum at single-cell resolution. Sci. Adv. 10, eadq6017 (2024). 39661664 10.1126/sciadv.adq6017 PMC11633743

[48] D. J. Ottenheimer, R. C. Simon, C. T. Burke, A. J. Bowen, S. M. Ferguson, G. D. Stuber, Single-cell sequencing of rodent ventral pallidum reveals diverse neuronal subtypes with non-canonical interregional continuity. bioRxiv 585611 [Preprint] (2024). 10.1101/2024.03.18.585611. PMC12674124 41337598

[49] C. P. Bengtson, P. B. Osborne, Electrophysiological properties of cholinergic and noncholinergic neurons in the ventral pallidal region of the nucleus basalis in rat brain slices. J. Neurophysiol. 83, 2649–2660 (2000). 10805665 10.1152/jn.2000.83.5.2649

[50] Y. M. Kupchik, P. W. Kalivas, The rostral subcommissural ventral pallidum is a mix of ventral pallidal neurons and neurons from adjacent areas: An electrophysiological study. Brain Struct. Funct. 218, 1487–1500 (2013). 23143342 10.1007/s00429-012-0471-9 PMC3600056

[51] B. Chieng, Y. Azriel, S. Mohammadi, M. J. Christie, Distinct cellular properties of identified dopaminergic and GABAergic neurons in the mouse ventral tegmental area. J. Physiol. (Lond.) 589, 3775–3787 (2011). 21646409 10.1113/jphysiol.2011.210807 PMC3171885

[52] P. Zhong, C. R. Vickstrom, X. Liu, Y. Hu, L. Yu, H.-G. Yu, Q.-S. Liu, HCN2 channels in the ventral tegmental area regulate behavioral responses to chronic stress. eLife 7, e32420 (2018). 29256865 10.7554/eLife.32420 PMC5749952

[53] L. Mu, X. Liu, H. Yu, C. R. Vickstrom, V. Friedman, T. J. Kelly, Y. Hu, W. Su, S. Liu, J. R. Mantsch, Q.-S. Liu, cAMP-mediated upregulation of HCN channels in VTA dopamine neurons promotes cocaine reinforcement. Mol. Psychiatry 28, 3930–3942 (2023). 37845497 10.1038/s41380-023-02290-x PMC10730389

[54] E. B. Margolis, B. Toy, P. Himmels, M. Morales, H. L. Fields, Identification of rat ventral tegmental area GABAergic neurons. PLOS ONE 7, e42365 (2012). 22860119 10.1371/journal.pone.0042365 PMC3409171

[55] S. Lammel, D. I. Ion, J. Roeper, R. C. Malenka, Projection-specific modulation of dopamine neuron synapses by aversive and rewarding stimuli. Neuron 70, 855–862 (2011). 21658580 10.1016/j.neuron.2011.03.025 PMC3112473

