# Supplementary material for: A ventral pallidal glutamatergic aversive network encodes abstinence from and reexposure to cocaine
Source: Sci Adv. 2025 Jul 23;11(30):eadu6074. doi: 10.1126/sciadv.adu6074 (PMC12285697; doi:10.1126/sciadv.adu6074)
Supplement: Supplementary file 1 — Supplementary Text Figs. S1 to S8 Tables S1 to S3 [file sciadv.adu6074_sm.pdf]

Science Advances
AAAS

Supplementary Materials for

A ventral pallidal glutamatergic aversive network encodes abstinence from and reexposure to cocaine

Liran A. Levi *et al.*

Corresponding author: Yonatan M. Kupchik, yonatan.kupchik@mail.huji.ac.il

Sci. Adv. **11**, eadu6074 (2025)
DOI: 10.1126/sciadv.adu6074

This PDF file includes:

Supplementary Text
Figs. S1 to S8
Tables S1 to S3

Supplementary Text

Supplementary Methods

CPP correction – We used here the unbiased CPP protocol, such that half the mice received cocaine in one side of the box and the rest in the other side of the box. In the behavioral experiments (Figs. 1,6) this does not affect the examination of the effect of VP_{Glu} inhibition on cocaine CPP as each mouse undergoes two tests and the examination is paired between these two tests. However, when correlating the electrophysiological changes with the CPP score (Fig. S7) we are interested in the net effect of the conditioning. For example, a mouse showing a CPP score of 0.3 that showed a-priori preference to the other side of the box (CPP score of -0.2 during habituation) would have a bigger net effect (0.5) than a mouse with the same CPP score that was indifferent to the sides before the test (0.3). To yield the net effect of the conditioning protocol in the electrophysiological experiments, we measured the CPP score twice – once during habituation (CPP_{Hab}) and once during the final test (CPP_{Test}). The net CPP score used in Fig. S7 was calculated by subtracting CPP_{Hab} from CPP_{Test}.

Fig. S1.

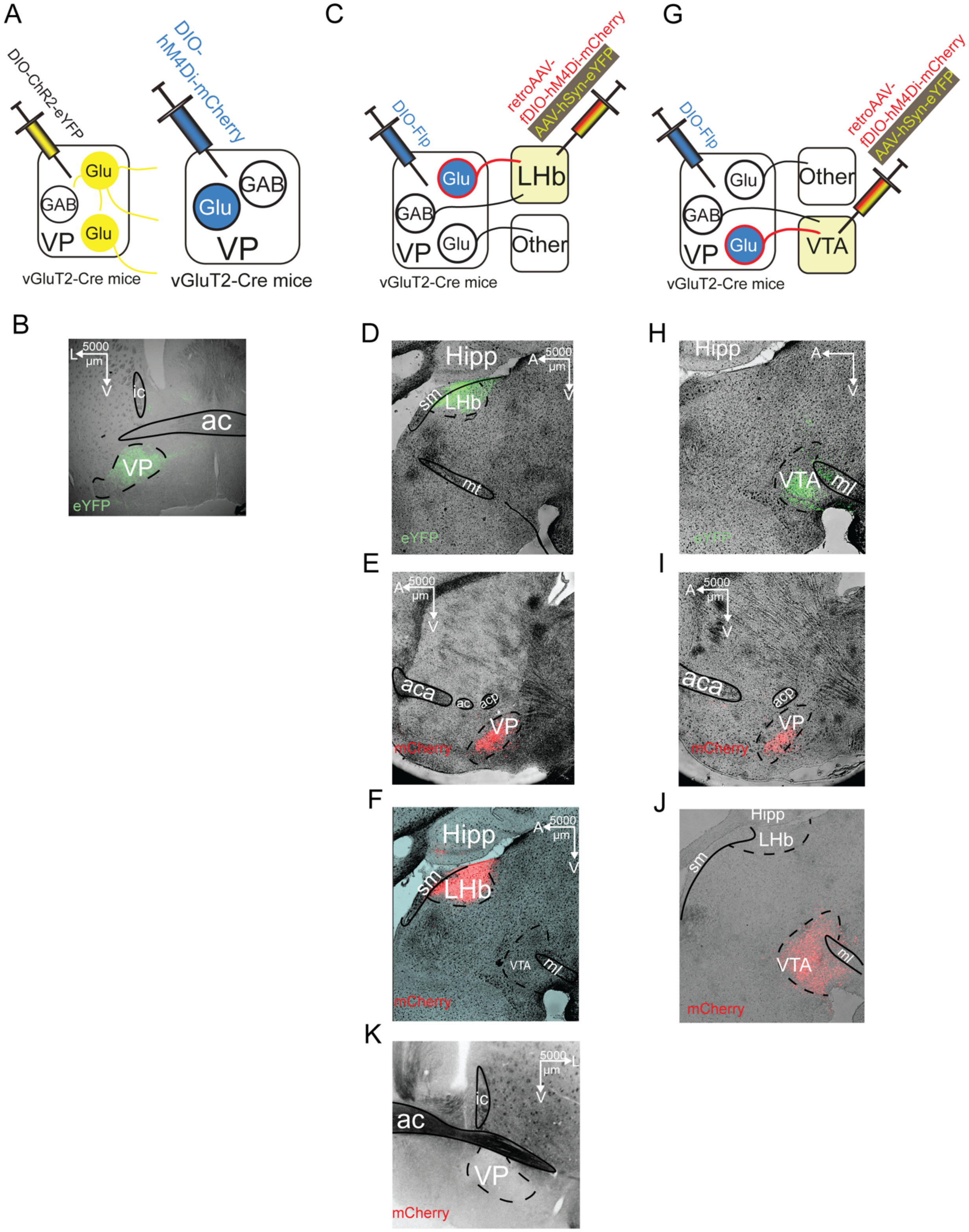
A
DIO-ChR2-eYFP
DIO-hM4Di-mCherry
Glu
GAB
VP
vGluT2-Cre mice
C
DIO-Flp
retroAAV-fDIO-hM4Di-mCherry
AAV-hSyn-eYFP
LHb
Other
G
VTA
B
ic
ac
eYFP
D
Hipp
sm
mt
E
aca
acp
mCherry
F
ml
H
I
J
K
5000 µm
L
V
A

Sites of injection in the VP, LHb and VTA. (**A-B**) Injections of AAV-DIO-ChR2-eYFP or AAV-DIO-h4Mdi-mCherry into the VP of vGluT2-Cre mice. (**A**) Injection setup. (**B**) Expression of AAV-DIO-ChR2-eYFP injected into the VP of a vGluT2-Cre mouse. Similar expression was achieved also with the injection of AAV-DIO-hM4Di-mCherry into the VP of vGluT2-Cre mice. (**C-F**) Injection of retroAAV-fDIO-hM4Di-mCherry to the LHb and AAV-DIO-Flp into the VP of vGluT2-Cre mice. The retroAAV-fDIO-hM4Di-mCherry was injected into the LHb together with AAV-hSyn-eYFP to verify the injection site. (**C**) Injection setup. (**D**) Expression of AAV-hSyn-eYFP in the LHb to verify injection site. (**E**) VP expression of the retroAAV-fDIO-hM4Di-mCherry that was injected in the LHb, indicating also successful injection of the AAV-DIO-Flp virus into the VP. (**F**) VP_{Glu} fibers expressing the retroAAV-fDIO-hM4Di-mCherry innervating the LHb, but not the VTA, when this virus was injected in the LHb and AAV-DIO-Flp was injected in the VP of vGluT2-Cre mice. (**G-J**) Injection of retroAAV-fDIO-hM4Di-mCherry to the VTA and AAV-DIO-Flp into the VP of vGluT2-Cre mice. The retroAAV-fDIO-hM4Di-mCherry was injected into the VTA together with AAV-hSyn-eYFP to verify the injection site. (**G**) Injection setup. (**H**) Expression of AAV-hSyn-eYFP in the VTA to verify injection site. (**I**) VP expression of the retroAAV-fDIO-hM4Di-mCherry that was injected in the VTA, indicating also successful injection of the AAV-DIO-Flp virus into the VP. (**J**) VP_{Glu} fibers expressing the retroAAV-fDIO-hM4Di-mCherry innervating the VTA, but not the LHb, when this virus was injected in the VTA and AAV-DIO-Flp was injected in the VP of vGluT2-Cre mice. (**K**) Performing the injections described in C or G on a WT mouse did not yield any expression of mCherry, thus ensuring the lack of non-specific expression. ac – anterior commissure; aca – anterior limb of anterior commissure; acp – posterior limb of anterior commissure; Hipp – hippocampus; ic – internal capsule; ml – medial lemniscus; mt – mammillothalamic tract; sm – stria medullaris of the thalamus

Fig. S2.

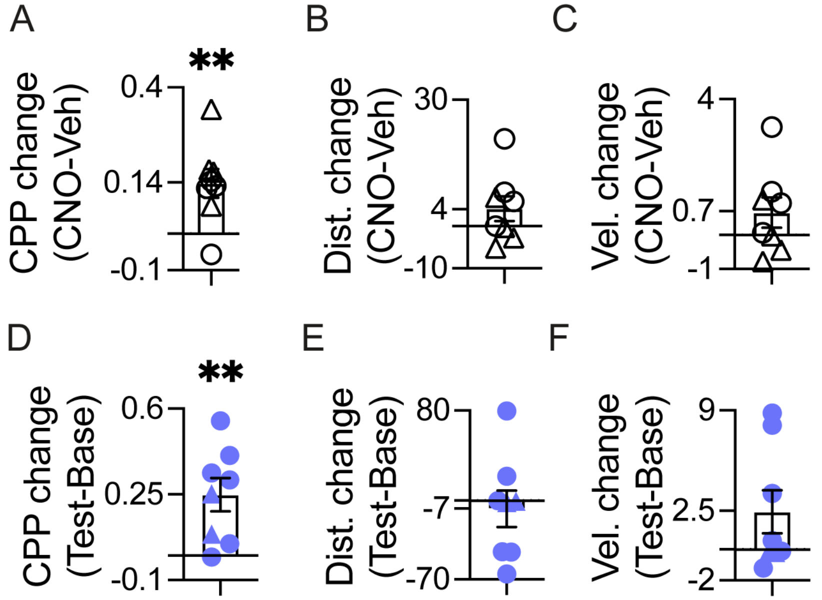

Average increase in the CPP score when conditioning to cocaine or VP$_{Glu}$ inhibition. Data taken from the same experiments performed in Fig. 1. (**A-C**) Cocaine conditioning. The change in CPP score, distance covered or velocity induced by inhibition of the VP$_{Glu}$ neurons during the CPP test was calculated by subtracting the performance in the control experiment (vehicle injection) from the performance after CNO injection. Inhibition of the VP$_{Glu}$ neurons during the CPP test caused a significant average increase of 0.14±0.11 in the (**A**) CPP score (one-sample t-test comparing to zero, t_7=3.53, p=0.0097) but did not affect the (**B**) distance covered (a change of 4.15±8.2 m, t_7=1.43 p=0.19) or the (**C**) velocity (a change of 0.65±1.3 cm/s, t_7=1.48 p=0.18). (**D-F**) Conditioning to the inhibition of VP$_{Glu}$ neurons. The change in CPP score, distance covered or velocity was calculated by subtracting the performance in the habituation test from the performance in the CPP test. (**D**) The CPP score was 0.25±0.19 points higher in the CPP test compared to the habituation (one-sample t-test comparing to zero, t_7=3.65, p=0.0082). The changes in distance covered (**E**) (a change of -7.16±45.9 m, t_7=0.44 p=0.67) and the velocity (**F**) (a change of 2.44±3.96 cm/s, t_7=1.74 p=0.13) were not significantly different from zero. Data presented as mean±SD, error bars represent s.e.m. Triangles – males. Circles – females.

Fig. S3.

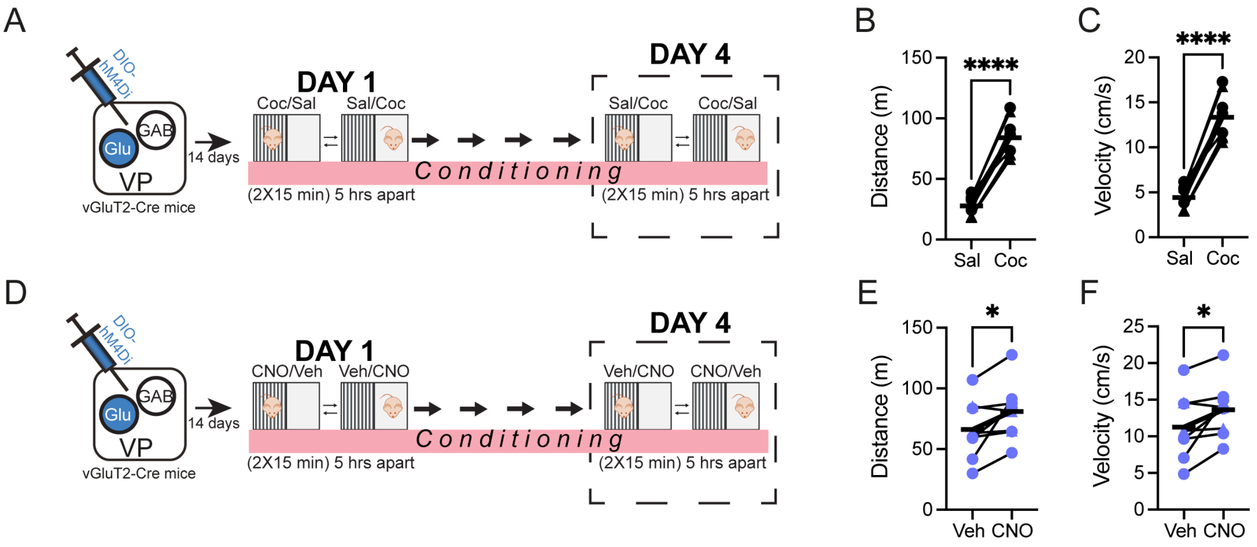

Both cocaine and inhibition of VP$_{Glu}$ neurons increase locomotion. Mice are the same as those used for Fig. 1. (A) Experimental protocol. vGluT2-Cre mice were first injected with a virus encoding for the inhibitory DREADD hM4Di in a cre-dependent manner (AAV-DIO-hM4Di) into the VP. Fourteen days later we conditioned a specific side of the CPP box to cocaine by injecting the mice alternately cocaine (15 mg/kg, i.p.) in one side of a two-chamber box or saline in the other side and leaving them in that side for 15 minutes. Sessions took place twice a day 5 hours apart for 4 consecutive days. (B,C,E,F) Circles – individual data. Solid lines – group averages. (B-C) Distance traveled (B) and average velocity (C) of each mouse during the two sessions of Day 4 (order of injections was counterbalanced). Cocaine caused a ~3-fold increase in both the distance (Paired t-test, t_8=13.6, p<0.0001) and velocity (Paired t-test, t_8=13.5, p<0.0001). (D) Experimental protocol. vGluT2-Cre mice were first injected with a virus encoding for the inhibitory DREADD hM4Di in a cre-dependent manner (AAV-DIO-hM4Di) into the VP. Fourteen days later we conditioned a specific side of the CPP box to the inhibition of VP$_{Glu}$ neurons by injecting CNO (3 mg/kg, i.p.) in that side and injecting vehicle in the other side. Mice underwent 2 such sessions per day, 5 hours apart (order of injections was counterbalanced), for 4 consecutive days. (E-F) Distance traveled (E) and average velocity (F) of each mouse during the two sessions of Day 4 (order of injections was counterbalanced). Inhibition of VP$_{Glu}$ neurons caused an increase of about 20% in both the distance (Paired t-test, t_8=2.75, p=0.025) and velocity (Paired t-test, t_8=2.75, p=0.025).
Type or paste caption here. Create a page break and paste in the Figure above the caption.

Fig. S4.

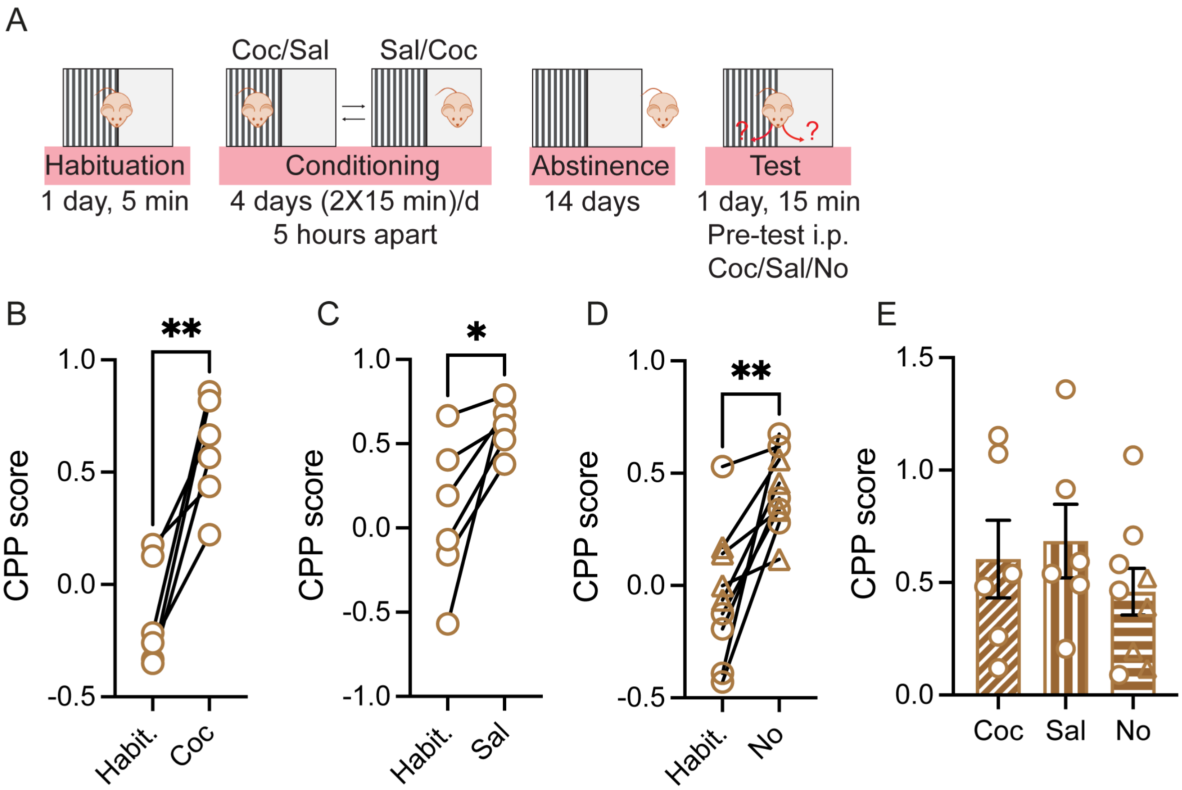

Re-introduction to the CPP box after 14 days of abstinence from cocaine generated place preference without and with pre-test injection of cocaine or saline. (**A**) Experimental protocol. Mice are the same as those in Fig. 2 used for electrophysiology experiments. One day after an habituation session, mice underwent cocaine CPP (4 days, two 15 min sessions per day 5 hours apart; mice received cocaine (15 mg/kg i.p.) on one side of the box and saline on the other; injection order counterbalanced), 14 days of abstinence and then re-exposure to the CPP box. The re-exposure to the CPP box was preceded by either an injection of cocaine (Coc, 15 mg/kg i.p.), an injection of saline (Sal) or no injection (No). (**B**) The CPP score increased from -0.14±0.23 in habituation to 0.59±0.24 in mice receving a cocaine injection prior to the CPP test (paired t-test, t_5=5.01, p=0.004). (**C**) The CPP score increased from 0.08±0.44 in habituation to 0.63±0.16 in mice receving a saline injection prior to the CPP test (paired t-test, t_5=3.07, p=0.03). (**D**) The CPP score increased from -0.04±0.30 in habituation to 0.42±0.18 in mice receiving a cocaine injection prior to the CPP test (paired t-test, t_8=4.42, p=0.002). (**E**) The average increase in the CPP score compared to the habituation was not affected by the pre-test treatment (One-way ANOVA, $F_{(2,18)}$=0.71, p=0.51).

Fig. S5.

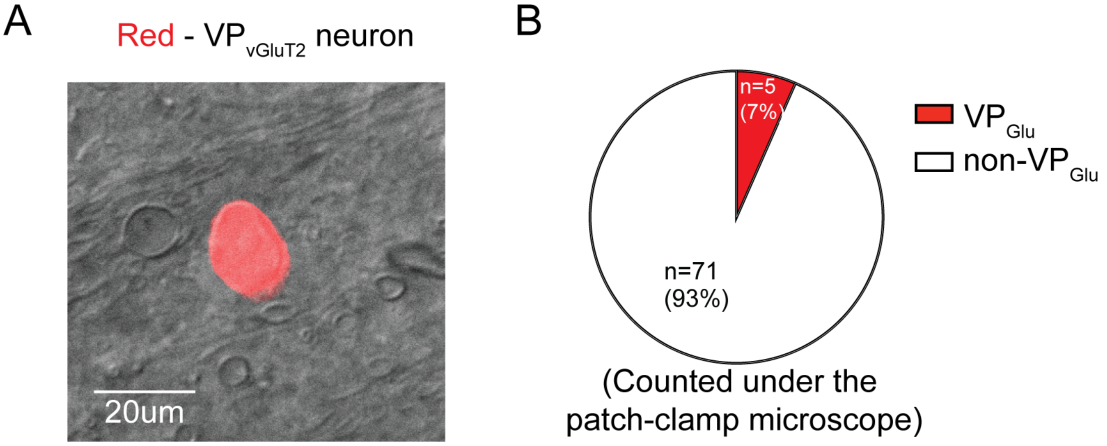

Labeled neurons in the VP of vGluT2-Cre X Ai9 mice make less than 10% of all neurons. (A) A representative micrograph of the VP of vGluT2-Cre X Ai9 mice with endogenous expression of tdTomato in neurons expressing (or that have expressed during development) vGluT2 as seen under the patch clamp microscope. (B) A pie chart depicting the number of neurons expressing or lacking tdTomato in the VP of vGluT2-Cre X Ai9 mice as seen under the patch-clamp microscope. All neurons were first detected in bright field mode and then tested for fluorescence. Note that the labeled neurons make approximately 7% of all neurons, in line with recent findings on VP_{Glu} neurons (REFs Stuber, Creed) and supporting the assumption that labeled neurons are indeed VP_{Glu} neurons.

Fig. S6.

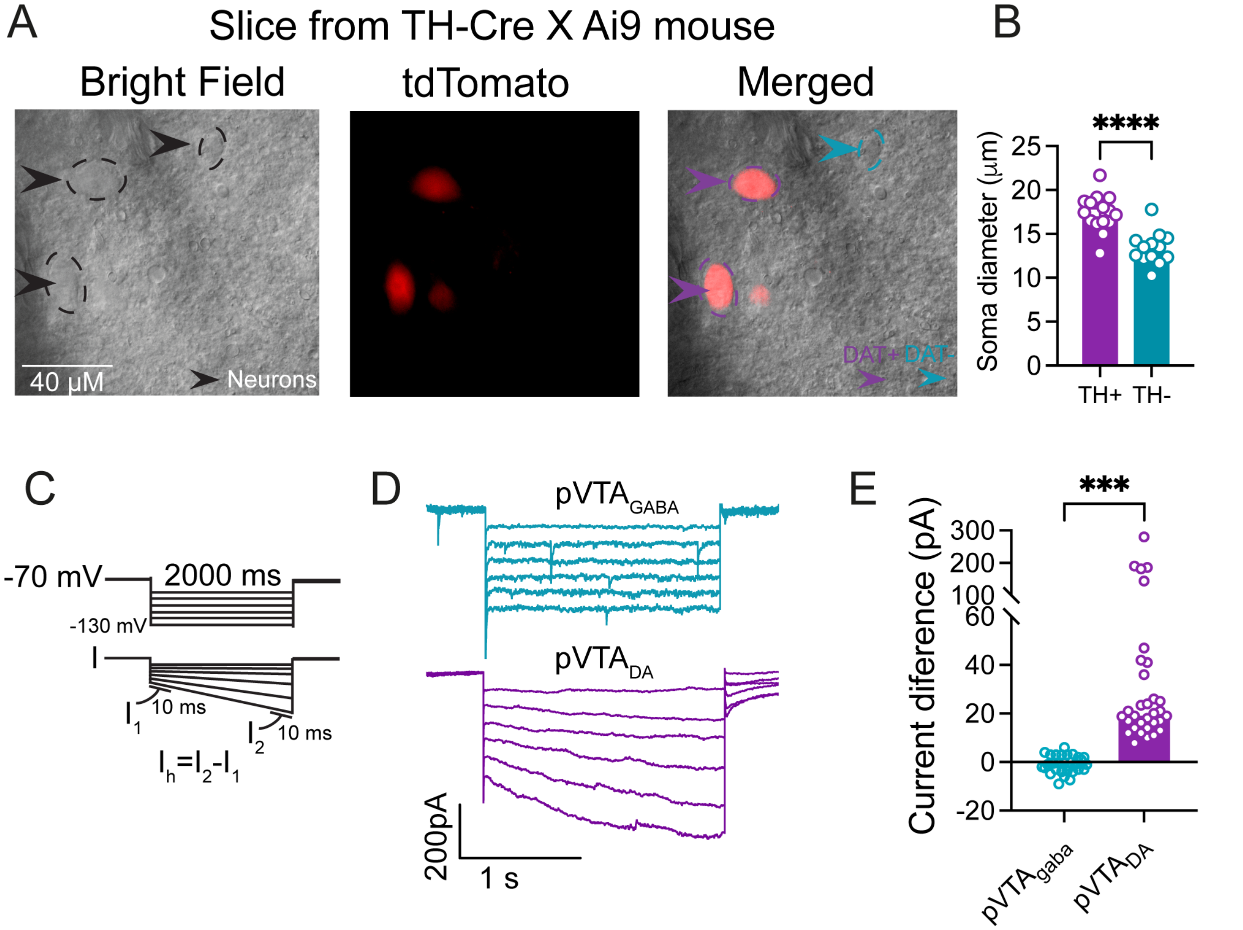

Identifying putative VTA_{DA} neurons. (**A-B**) Putative VTA_{DA} neurons have bigger soma sizes. (**A**) An acute VTA slice taken from a TH-Cre X Ai9 mouse, which expresses tdTomato in neurons expressing the enzyme tyrosine hydroxylase (TH), a marker of dopaminergic neurons in the VTA. Left – bright field image. Arrows point to identified neurons. Middle – tdTomato fluorescence by TH-expressing neurons. Right – Merge. Two of the identified VTA neurons express TH (purple arrows) while the other does not (cyan arrow). (**B**) The average soma diameter of TH-expressing neurons (17.4±2.1 μm) was significantly bigger than that of VTA neurons not expressing TH (13.4±1.8 μm) (Unpaired t-test, t_{26}=5.42, p<0.0001). Diameters were measured at the widest part of the soma. (**C-E**) $pVTA_{DA}$ neurons express bigger Ih currents. (**C**) Experimental protocol. Neurons were voltage clamped at -70mV and six 500 ms-long hyperpolarizing steps of -10, -20, -30, -40, -50 and -60 mV were applied sequentially (3 sec inter-pulse interval). Currents were recorded at each step and the average amplitudes of the currents at the first (I_1) and last (I_2) 10 ms of the pulse were calculated. The I_h current was calculated by subtracting I_2 from I_1. (**D**) Representative I_h currents recorded from $pVTA_{GABA}$ (top) and $pVTA_{DA}$ (bottom) neurons. (**E**) The average I_h current of $pVTA_{DA}$ neurons (48.2±67.9 pA) was significantly bigger than that of $pVTA_{GABA}$ neurons (-0.98±3.61 pA) (unpaired t-test, t_{59}=3.90, p=0.0003). Data taken from same cells recorded in Figures 2-4 of main text.

Fig. S7.

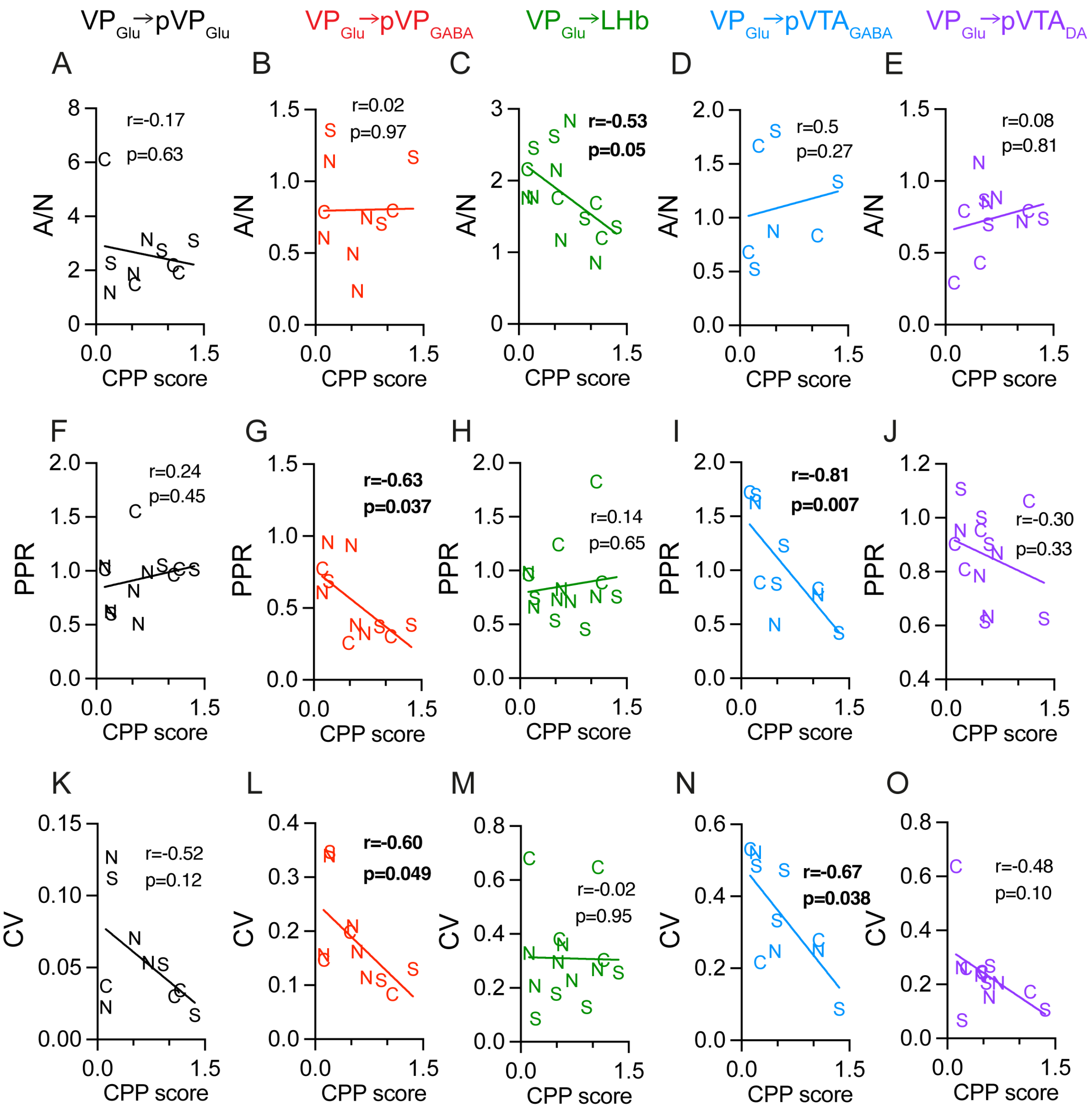

Correlations between the CPP score and synaptic parameters in various synapses of VP_{Glu} neurons. The CPP scores were corrected normalized to the pre-conditioning preference (CPP_{Test} – $CPP_{Habituation}$) from all conditions (pre-test cocaine injection (C), pre-test saline injection (S) and no pre-test injection (N)) are pooled together. The A/N, PPR and CV for each mouse represents the average value from all recorded neurons in this mouse (in a specific projection). (A-E) A/N. (F-J) PPR. (K-O) CV. The only synapse showing a significant correlation of the CPP score with A/N is the VP_{Glu}→LHb synapse, showing an inverse correlation (C). Two synapses showed a significant inverse correlation between the CPP score and both the PPR and the CV – the VP_{Glu}→pVP_{GABA} (G,L) and VP_{Glu}→$pVTA_{GABA}$ (I,N). r- Pearson's r. p values were calculated from an F-test examining in each graph whether the slope of the regression line is equal to zero.

Fig. S8.

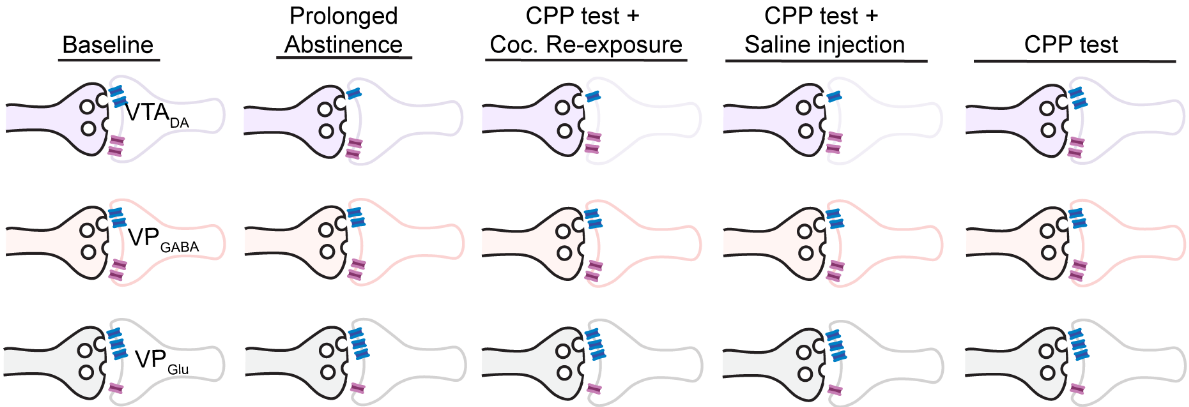

Graphical summary of the overall synaptic plasticity occurring in VP_{Glu}→$pVTA_{DA}$, VP_{Glu}→pVP_{GABA} and VP_{Glu}→pVP_{Glu} synapses. Number of vesicles represents presynaptic strength, number of blue (AMPA) receptors represents postsynaptic strength. Pink receptors – NMDA receptors. Color intensity represents overall synaptic strength. The synapses of the VP_{Glu} neurons on $pVTA_{DA}$, pVP_{GABA} or pVP_{Glu} neurons did not show significant overall plasticity driven by either condition tested here.

Table S1.

Figure	Gi-DREADD expression	Comparison	Measurement	Veh. / Baseline (males/females)	CNO / Test (males/females)	Statistical test	Statistic	P value
Figure 1C	VPglu	Vehicle vs CNO	CPP score	0.23±0.19 (4/4)	0.37±0.20 (4/4)	Paired t-test	t(7)=3.53	**p=0.0097**
Figure 1E			Distance	43.7±5.8 (4/4)	47.9±10.3 (4/4)	Paired t-test	t(7)=1.43	p=0.195
Figure 1F			Velocity	6.96±0.94 (4/4)	7.62±1.62 (4/4)	Paired t-test	t(7)=1.48	p=0.182
Figure 1H	VPglu	Test vs zero	CPP score	-	-	One-sample t-test	t(7)=5.93	**p=0.0006**
Figure 1H		Baseline vs Test	CPP score	-0.05±0.15 (2/6)	0.20±0.09 (2/6)	Paired t-test	t(7)=3.65	**p=0.0082**
Figure 1J			Distance	145.3±41.6 (2/6)	138.1±28.7 (2/6)	Paired t-test	t(7)=0.44	p=0.6724
Figure 1K			Velocity	16.9±4.3 (2/6)	19.3±3.9 (2/6)	Paired t-test	t(7)=1.74	p=0.1257
Figure 7C	VPglu-->LHb	Vehicle vs CNO	CPP score	0.15±0.07 (3/3)	0.31±0.19 (3/3)	Paired t-test	t(5)=2.61	**p=0.031**
Figure 7E			Distance	64.4±11.1 (3/3)	55.1±18.2 (3/3)	Paired t-test	t(5)=1.12	p=0.314
Figure 7F			Velocity	7.17±1.24 (3/3)	6.13±2.02 (3/3)	Paired t-test	t(5)=1.12	p=0.311
Figure 7I	VPglu-->VTA	Vehicle vs CNO	CPP score	0.29±0.19 (2/4)	0.27±0.17 (2/4)	Paired t-test	t(5)=0.208	p=0.844
Figure 7K			Distance	55.4±4.7 (2/4)	49.4±7.5 (2/4)	Paired t-test	t(5)=1.89	p=0.118
Figure 7L			Velocity	6.17±0.52 (2/4)	5.50±0.84 (2/4)	Paired t-test	t(5)=1.90	p=0.116

Statistical data for the behavioral experiments presented in figures 1 and 6. Bold fonts highlight comparisons that show significant differences between groups. Numbers represent the means ± sd

Table S2.

Figure	Parameter	VP_{Glu}→	Effect of condition (all compared to 14d abstinence)				Differences between CPP pre-test treatments			
Figure 2C	A/N	LHb	Main condition effect	1-way ANOVA	F(4,34)=4.70	**p=0.004**	Main treatment effect	1-way ANOVA	F(2,22)=1.09	p=0.355
			1d vs 14d	Dunnett's MCT	q(34)=3.89	**p=0.002**	Coc vs Sal	Tukey's MCT	q(22)=2.08	p=0.323
			Coc vs 14d	Dunnett's MCT	q(34)=3.24	**p=0.009**	Coc vs No inj.	Tukey's MCT	q(22)=1.02	p=0.753
			Sal vs 14d	Dunnett's MCT	q(34)=1.61	p=0.333	Sal vs No inj.	Tukey's MCT	q(22)=1.22	p=0.669
			No inj. vs 14d	Dunnett's MCT	q(34)=2.33	p=0.04				
Figure 2D		$pVTA_{DA}$	Main condition effect	1-way ANOVA	F(4,29)=5.24	**p=0.003**	Main treatment effect	1-way ANOVA	F(2,19)=3.26	p=0.061
			1d vs 14d	Dunnett's MCT	q(29)=3.65	**p=0.004**	Coc vs Sal	Tukey's MCT	q(19)=0.86	p=0.817
			Coc vs 14d	Dunnett's MCT	q(29)=0.72	p=0.886	Coc vs No inj.	Tukey's MCT	q(19)=3.45	p=0.061
			Sal vs 14d	Dunnett's MCT	q(29)=1.40	p=0.450	Sal vs No inj.	Tukey's MCT	q(19)=2.70	p=0.163
			No inj. vs 14d	Dunnett's MCT	q(29)=3.35	**p=0.008**				
Figure 2E		$pVTA_{GABA}$	Main condition effect	1-way ANOVA	F(4,26)=1.55	p=0.217	Main treatment effect	1-way ANOVA	F(2,13)=0.01	p=0.992
			1d vs 14d	Dunnett's MCT	q(26)=0.21	p=0.999	Coc vs Sal	Tukey's MCT	q(13)=0.18	p=0.991
			Coc vs 14d	Dunnett's MCT	q(26)=1.59	p=0.363	Coc vs No inj.	Tukey's MCT	q(13)=0.08	p=0.998
			Sal vs 14d	Dunnett's MCT	q(26)=1.62	p=0.348	Sal vs No inj.	Tukey's MCT	q(13)=0.08	p=0.998
			No inj. vs 14d	Dunnett's MCT	q(26)=1.42	p=0.467				
Figure 2F		pVP_{GABA}	Main condition effect	1-way ANOVA	F(4,28)=1.35	p=0.278	Main treatment effect	1-way ANOVA	F(2,16)=1.78	p=0.200
			1d vs 14d	Dunnett's MCT	q(28)=0.18	p=0.999	Coc vs Sal	Tukey's MCT	q(16)=1.76	p=0.448
			Coc vs 14d	Dunnett's MCT	q(28)=0.77	p=0.866	Coc vs No inj.	Tukey's MCT	q(16)=0.62	p=0.900
			Sal vs 14d	Dunnett's MCT	q(28)=2.13	p=0.137	Sal vs No inj.	Tukey's MCT	q(16)=2.63	p=0.184
			No inj. vs 14d	Dunnett's MCT	q(28)=0.41	p=0.984				
Figure 2G		pVP_{Glu}	Main condition effect	1-way ANOVA	F(4,33)=2.76	p=0.044	Main treatment effect	1-way ANOVA	F(2,19)=2.17	p=0.142
			1d vs 14d	Dunnett's MCT	q(33)=0.84	p=0.831	Coc vs Sal	Tukey's MCT	q(19)=2.85	p=0.135
			Coc vs 14d	Dunnett's MCT	q(33)=0.96	p=0.757	Coc vs No inj.	Tukey's MCT	q(19)=1.97	p=0.364
			Sal vs 14d	Dunnett's MCT	q(33)=2.90	p=0.024	Sal vs No inj.	Tukey's MCT	q(19)=1.03	p=0.751
			No inj. vs 14d	Dunnett's MCT	q(33)=2.36	p=0.082				
Figure 3A	PPR	LHb	Main condition effect	1-way ANOVA	F(4,35)=4.85	**p=0.003**	Main treatment effect	1-way ANOVA	F(2,23)=7.86	**p=0.003**
			1d vs 14d	Dunnett's MCT	q(35)=1.75	p=0.26	Coc vs Sal	Tukey's MCT	q(23)=5.05	**p=0.004**
			Coc vs 14d	Dunnett's MCT	q(35)=3.95	**p=0.001**	Coc vs No inj.	Tukey's MCT	q(23)=4.52	**p=0.01**
			Sal vs 14d	Dunnett's MCT	q(35)=0.31	p=0.994	Sal vs No inj.	Tukey's MCT	q(23)=1.25	p=0.658
			No inj. vs 14d	Dunnett's MCT	q(35)=1.25	p=0.541				
Figure 3B		$pVTA_{DA}$	Main condition effect	1-way ANOVA	F(4,34)=0.45	p=0.773	Main treatment effect	1-way ANOVA	F(2,24)=0.60	p=0.556
			1d vs 14d	Dunnett's MCT	q(34)=0.95	p=0.743	Coc vs Sal	Tukey's MCT	q(24)=1.54	p=0.529
			Coc vs 14d	Dunnett's MCT	q(34)=0.95	p=0.743	Coc vs No inj.	Tukey's MCT	q(24)=0.85	p=0.821
			Sal vs 14d	Dunnett's MCT	q(34)=0.09	p>0.999	Sal vs No inj.	Tukey's MCT	q(24)=0.55	p=0.920
			No inj. vs 14d	Dunnett's MCT	q(34)=0.39	p=0.984				

Figure 3C		$pVTA_{GABA}$	Main condition effect	1-way ANOVA	F(4,29)=4.22	**p=0.008**	Main treatment effect	1-way ANOVA	F(2,16)=2.19	p=0.145
			1d vs 14d	Dunnett's MCT	q(29)=2.38	p=0.082	Coc vs Sal	Tukey's MCT	q(16)=1.00	p=0.764
			Coc vs 14d	Dunnett's MCT	q(29)=3.71	**p=0.003**	Coc vs No inj.	Tukey's MCT	q(16)=2.93	p=0.127
			Sal vs 14d	Dunnett's MCT	q(29)=2.68	p=0.042	Sal vs No inj.	Tukey's MCT	q(16)=1.86	p=0.406
			No inj. vs 14d	Dunnett's MCT	q(29)=1.02	p=0.727				
Figure 3D		pVP_{GABA}	Main condition effect	1-way ANOVA	F(4,32)=1.71	p=0.173	Main treatment effect	1-way ANOVA	F(2,18)=0.46	p=0.636
			1d vs 14d	Dunnett's MCT	q(32)=1.27	p=0.533	Coc vs Sal	Tukey's MCT	q(18)=0.43	p=0.95
			Coc vs 14d	Dunnett's MCT	q(32)=0.86	p=0.811	Coc vs No inj.	Tukey's MCT	q(18)=0.96	p=0.777
			Sal vs 14d	Dunnett's MCT	q(32)=1.13	p=0.634	Sal vs No inj.	Tukey's MCT	q(18)=1.32	p=0.629
			No inj. vs 14d	Dunnett's MCT	q(32)=0.09	p>0.999				
Figure 3E		pVP_{Glu}	Main condition effect	1-way ANOVA	F(4,39)=2.57	p=0.053	Main treatment effect	1-way ANOVA	F(2,25)=0.46	p=0.636
			1d vs 14d	Dunnett's MCT	q(39)=1.49	p=0.390	Coc vs Sal	Tukey's MCT	q(18)=0.43	p=0.95
			Coc vs 14d	Dunnett's MCT	q(39)=3.08	**p=0.014**	Coc vs No inj.	Tukey's MCT	q(18)=0.96	p=0.777
			Sal vs 14d	Dunnett's MCT	q(39)=1.31	p=0.503	Sal vs No inj.	Tukey's MCT	q(18)=1.32	p=0.629
			No inj. vs 14d	Dunnett's MCT	q(39)=0.96	p=0.743				
Figure 4A		LHb	Main condition effect	1-way ANOVA	F(4,35)=7.13	**p<0.001**	Main treatment effect	1-way ANOVA	F(2,23)=7.03	**p=0.004**
			1d vs 14d	Dunnett's MCT	q(35)=3.86	**p=0.002**	Coc vs Sal	Tukey's MCT	q(23)=5.14	**p=0.004**
			Coc vs 14d	Dunnett's MCT	q(35)=4.32	**p<0.001**	Coc vs No inj.	Tukey's MCT	q(23)=3.57	**p=0.01**
			Sal vs 14d	Dunnett's MCT	q(35)=0.50	p=0.967	Sal vs No inj.	Tukey's MCT	q(23)=2.18	p=0.292
			No inj. vs 14d	Dunnett's MCT	q(35)=2.19	p=0.114				
Figure 4B		$pVTA_{DA}$	Main condition effect	1-way ANOVA	F(4,34)=1.76	p=0.159	Main treatment effect	1-way ANOVA	F(2,24)=1.21	p=0.316
			1d vs 14d	Dunnett's MCT	q(34)=2.04	p=0.148	Coc vs Sal	Tukey's MCT	q(24)=2.16	p=0.295
			Coc vs 14d	Dunnett's MCT	q(34)=1.22	p=0.555	Coc vs No inj.	Tukey's MCT	q(24)=1.34	p=0.617
			Sal vs 14d	Dunnett's MCT	q(34)=0.14	p=0.999	Sal vs No inj.	Tukey's MCT	q(24)=0.62	p=0.899
			No inj. vs 14d	Dunnett's MCT	q(34)=0.27	p=0.996				
Figure 4C	CV	$pVTA_{GABA}$	Main condition effect	1-way ANOVA	F(4,29)=4.85	**p=0.005**	Main treatment effect	1-way ANOVA	F(2,16)=0.56	p=0.582
			1d vs 14d	Dunnett's MCT	q(29)=2.94	**p=0.014**	Coc vs Sal	Tukey's MCT	q(16)=0.189	p=0.990
			Coc vs 14d	Dunnett's MCT	q(29)=3.83	**p=0.003**	Coc vs No inj.	Tukey's MCT	q(16)=1.43	p=0.581
			Sal vs 14d	Dunnett's MCT	q(29)=3.29	**p=0.010**	Sal vs No inj.	Tukey's MCT	q(16)=1.15	p=0.701
			No inj. vs 14d	Dunnett's MCT	q(29)=2.17	p=0.129				
Figure 4D		pVP_{GABA}	Main condition effect	1-way ANOVA	F(4,32)=1.44	p=0.243	Main treatment effect	1-way ANOVA	F(2,18)=1.38	p=0.277
			1d vs 14d	Dunnett's MCT	q(32)=0.86	p=0.813	Coc vs Sal	Tukey's MCT	q(18)=1.86	p=0.405
			Coc vs 14d	Dunnett's MCT	q(32)=2.33	p=0.086	Coc vs No inj.	Tukey's MCT	q(18)=2.12	p=0.315
			Sal vs 14d	Dunnett's MCT	q(32)=0.77	p=0.861	Sal vs No inj.	Tukey's MCT	q(18)=0.17	p=0.993
			No inj. vs 14d	Dunnett's MCT	q(32)=0.67	p=0.909				
Figure 4E		pVP_{Glu}	Main condition effect	1-way ANOVA	F(4,35)=2.02	p=0.113	Main treatment effect	1-way ANOVA	F(2,25)=2.02	p=0.156
			1d vs 14d	Dunnett's MCT	q(35)=0.62	p=0.927	Coc vs Sal	Tukey's MCT	q(18)=1.79	p=0.428

			Coc vs 14d	Dunnett's MCT	q(35)=2.64	p=0.041	Coc vs No inj.	Tukey's MCT	q(18)=2.83	p=0.135
			Sal vs 14d	Dunnett's MCT	q(35)=1.69	p=0.285	Sal vs No inj.	Tukey's MCT	q(18)=0.85	p=0.821
			No inj. vs 14d	Dunnett's MCT	q(35)=1.42	p=0.430				

Data for all statistical tests performed in the electrophysiological experiments of figures 2-4. Bold fonts highlight comparisons that show significant differences between groups. MCT – Multiple comparisons test.

Table S3.

Figure	Parameter	Projection	Abstinence days		Pre-test injection		
			1d	14d	Cocaine	Saline	No
Fig. 2C	A/N	$VP_{Glu} \rightarrow LHb$	0.94±0.53 (5/3)	2.94±1.30 (9/5)	1.53±0.56 (9/4)	2.16±0.52 (6/4)	1.80±1.07 (10/6)
Fig. 2D		$VP_{Glu} \rightarrow pVTA_{DA}$	1.17±0.32 (5/2)	0.55±0.17 (7/4)	0.66±0.31 (7/3)	0.76±0.16 (8/3)	1.07±0.44 (7/4)
Fig. 2E		$VP_{Glu} \rightarrow pVTA_{GABA}$	0.68±0.20 (6/3)	0.74±0.22 (9/6)	1.14±0.65 (7/3)	1.20±0.71 (5/3)	1.17±0.70 (4/2)
Fig. 2F		$VP_{Glu} \rightarrow pVP_{GABA}$	0.74±0.56 (6/3)	0.70±0.29 (8/7)	0.87±0.21 (5/2)	1.15±0.37 (6/3)	0.78±0.44 (8/5)
Fig. 2G		$VP_{Glu} \rightarrow pVP_{Glu}$	1.73±1.44 (7/4)	1.33±0.29 (9/5)	1.77±0.56 (8/4)	2.77±0.79 (6/3)	2.41±1.24 (8/3)
Fig. 3A	PPR	$VP_{Glu} \rightarrow LHb$	0.87±0.49 (6/2)	0.58±0.20 (8/4)	1.18±0.43 (9/4)	0.63±0.17 (6/4)	0.76±0.18 (11/6)
Fig. 3B		$VP_{Glu} \rightarrow pVTA_{DA}$	0.91±0.49 (5/3)	0.75±0.27 (7/4)	0.89±0.26 (10/4)	0.76±0.27 (10/5)	0.81±0.23 (7/4)
Fig. 3C		$VP_{Glu} \rightarrow pVTA_{GABA}$	1.06±0.47 (6/3)	0.55±0.16 (9/6)	1.32±0.59 (7/3)	1.13±0.51 (6/4)	0.77±0.21 (6/3)
Fig. 3D		$VP_{Glu} \rightarrow pVP_{GABA}$	0.83±0.22 (8/3)	0.65±0.27 (8/7)	0.53±0.30 (8/3)	0.48±0.24 (6/3)	0.64±0.36 (7/5)
Fig. 3E		$VP_{Glu} \rightarrow pVP_{Glu}$	0.93±0.32 (7/4)	0.74±0.25 (9/5)	1.12±0.32 (9/4)	0.91±0.24 (7/3)	0.85±0.19 (12/5)
Fig. 4A	CV	$VP_{Glu} \rightarrow LHb$	0.449±0.259 (5/2)	0.142±0.080 (9/5)	0.432±0.198 (9/4)	0.179±0.082 (6/4)	0.282±0.072 (11/6)
Fig. 4B		$VP_{Glu} \rightarrow pVTA_{DA}$	0.370±0.194 (5/3)	0.197±0.116 (7/4)	0.284±0.199 (10/4)	0.187±0.089 (10/5)	0.218±0.096 (7/4)
Fig. 4C		$VP_{Glu} \rightarrow pVTA_{GABA}$	0.303±0.157 (6/3)	0.088±0.022 (9/6)	0.356±0.179 (7/3)	0.343±0.225 (5/3)	0.256±0.032 (5/3)
Fig. 4D		$VP_{Glu} \rightarrow pVP_{GABA}$	0.197±0.105 (8/3)	0.237±0.060 (8/7)	0.129±0.057 (8/3)	0.198±0.133 (6/3)	0.205±0.101 (7/5)
Fig. 4E		$VP_{Glu} \rightarrow pVP_{Glu}$	0.088±0.058 (7/4)	0.106±0.081 (8/5)	0.033±0.008 (7/3)	0.059±0.050 (7/3)	0.070±0.042 (11/5)

Means and standard deviations of all values in the electrophysiology experiments. Numbers in parentheses represent the number of recorded cells / number of mice from which these cells were recorded.
